# Zdhhc14 promotes pyroptosis in the dorsal root ganglion of oxaliplatin‐induced peripheral neuropathy mice by enhancing GBP5 palmitoylation and suppressing its ubiquitination

**DOI:** 10.1002/ctm2.70874

**Published:** 2026-11-04

**Authors:** Xiao Jiang, Wanqiu Li, Yanzhuo Liu, Wei Song, Xuedan Zheng, Liu Tang

**Affiliations:** ^1^ Department of Pharmacy Renmin Hospital of Wuhan University Wuhan Hubei China; ^2^ Department of Neurology, Suizhou Hospital Hubei University of Medicine Suizhou Hubei China; ^3^ Department of Neurology, The Sixth Hospital of Wuhan Affiliated Hospital of Jianghan University Wuhan Hubei China

**Keywords:** GBP5, oxaliplatin‐induced peripheral neuropathy, palmitoylation, pyroptosis, Zdhhc14

## Abstract

**Background:**

Oxaliplatin‐related neuropathy (OIPN) remains a frequent chemotherapy‐induced complication, yet its underlying mechanisms remain incompletely understood. Emerging evidence implicates pyroptosis in OIPN pathogenesis, and palmitoylation has been recognised as a critical mechanism in neurological disorders.

**Objective:**

This investigation set out to examine the part played by the palmitoyltransferase Zdhhc14 in pyroptosis in OIPN as well as to clarify the fundamental molecular processes.

**Methods:**

A mouse model of OIPN was developed through intraperitoneal oxaliplatin administration, and primary neuronal cultures derived from dorsal root ganglia were exposed to oxaliplatin to simulate OIPN in vitro. Bioinformatics analysis was performed using the GSE255097 dataset. Gene and protein expression were examined by quantitative real‐time PCR, Western blot and immunofluorescence staining. Pyroptosis was assessed by lactate dehydrogenase release, flow cytometry and detection of pyroptosis‐related proteins. Co‐immunoprecipitation, immunoprecipitation and cycloheximide chase assay were applied to evaluate the mechanisms.

**Results:**

Zdhhc14 was significantly up‐regulated in dorsal root ganglion (DRG) of OIPN mice in a time‐dependent manner. Zdhhc14 deficiency alleviated OIPN phenotypes, including mechanical hyperalgesia and cold allodynia, neuroinflammation and DRG pyroptosis. Mechanistically, Zdhhc14 binds to GBP5 and promotes its modification at Cys12, which is required for Zdhhc14‐mediated suppression of K48‐linked ubiquitination and subsequent proteasomal degradation, thereby stabilising GBP5. GBP5 knockdown phenocopied the protective effects of Zdhhc14 deficiency, whereas GBP5 overexpression reversed these effects. Importantly, mutation of the GBP5 palmitoylation site abolished the pro‐pyroptotic effects of Zdhhc14.

**Conclusions:**

These findings indicate that the Zdhhc14/GBP5 palmitoylation axis critically regulates DRG pyroptosis and neuroinflammation in OIPN, identifying a novel mechanism and a potential mechanistic target warranting further translational evaluation for this debilitating condition.

## Graphical abstract

Oxaliplatin‐induced up‐regulation of Zdhhc14 enhances GBP5 stability through palmitoylation‐dependent inhibition of ubiquitin–proteasome degradation, thereby contributing to the development of peripheral neuropathy.

Zdhhc14 is identified as a key palmitoyltransferase up‐regulated in oxaliplatin‐induced peripheral neuropathy.

Zdhhc14 mediates GBP5 palmitoylation at Cys12, promoting GSDMD‐dependent pyroptosis in DRG neurons.

Genetic ablation of Zdhhc14 or GBP5 protects against L‐OHP‐induced neuropathic pain

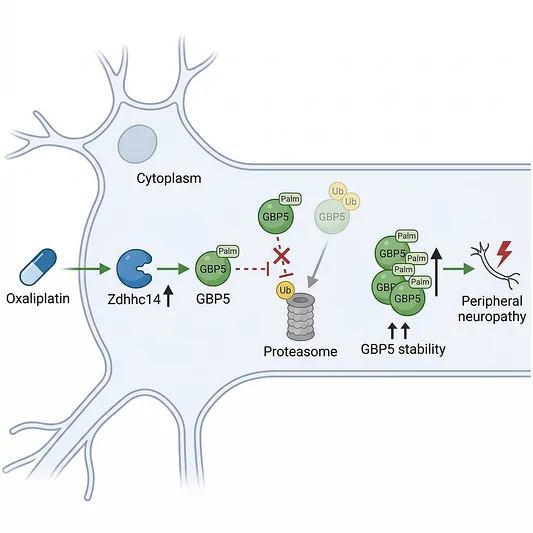

## 1 INTRODUCTION

Oxaliplatin, commonly known as L‑OHP, is a third‑generation platinum‑derived cytotoxic drug that sees regular application against gastrointestinal neoplasms, notably those arising from the colorectum or gastric mucosa.^1^, ^2^ Despite its clinical efficacy, L‐OHP is associated with dose‐limiting neurotoxicity, manifesting as acute and chronic peripheral neuropathy.^3^ Among these, L‐OHP‐induced peripheral neuropathy (OIPN) represents the most frequent and serious untoward reaction. Clinically, OIPN is characterised by sensory abnormalities, often accompanied by cold‐induced dysesthesia.^4^, ^5^ Such adverse events commonly mandate tapering or therapy cessation, thereby eroding efficacy and markedly diminishing patients’ QoL.^6^

Ion‑channel dysregulation, oxidative burden, neuroinflammatory responses and injury to dorsal root ganglion (DRG) neurons collectively underpin the notably multifaceted aetiology of OIPN.^7^, ^8^, ^9^ Neuroinflammation acts as a key pathogenic mediator in OIPN, marked by cytokine buildup and immune‐cell infiltration into peripheral neural tissues.^10^, ^11^ Pyroptosis, a pro‑inflammatory variant of regulated cell death, is strongly associated with immune activation. Its initiation relies on caspase‑1‑mediated proteolysis, which promotes pore formation and cytokine release (IL‑1β and IL‑18), consequently heightening the inflammatory milieu.^12^, ^13^ Pyroptosis is clearly implicated in OIPN progression in animals, yet the exact signalling routes linking its regulation to disease pathology are still poorly understood.^14^, ^15^

As a dynamic lipidation, palmitoylation reversibly links palmitic acid to proteinaceous cysteines, thereby steering their membrane docking, metabolic stability, intermolecular contacts and overall bioactivity.^16^, ^17^ Accumulating evidence has demonstrated that palmitoylation participates extensively in the etiopathogenesis of neurologic disorders.^18^, ^19^, ^20^ Notably, emerging studies have implicated palmitoylation in peripheral neuropathy, with dysregulation of palmitoylating enzymes and their substrates observed in injured peripheral nerves.^21^, ^22^ The zinc finger DHHC‐type containing 14 (Zdhhc14) is a member of the DHHC palmitoyltransferase family responsible for catalysing protein palmitoylation.^23^ Nonetheless, Zdhhc14's neural roles are largely obscure, and its OIPN‑related functions and mechanistic basis remain largely unexplored.

In this study, we established an L‐OHP‐induced OIPN mouse model and investigated the expression of Zdhhc14 in this animal model, as well as the mechanism by which Zdhhc14 regulates pyroptosis in OIPN mice through palmitoylation. Our work uncovers an unexpected involvement of Zdhhc14‐dependent palmitoylation in OIPN and suggests that this pathway may represent a mechanistic target for further preclinical investigation, although its therapeutic applicability remains to be established.

## 2 METHODS

### 2.1 Bioinformatic analysis

The public gene expression dataset GSE255097 was downloaded from the NCBI Gene Expression Omnibus (GEO; https://www.ncbi.nlm.nih.gov/geo/query/acc.cgi?acc=GSE255097). This dataset contains high‐throughput RNA sequencing profiles of mouse sciatic nerve blood vessels derived from L‐OHP‐treated and sham control C57BL/6 mice. The dataset comprises 12 samples in total: six biological replicates from L‐OHP‐treated mice (3 mg/kg, intraperitoneal injection for 5 consecutive days) and six biological replicates from sham control mice (saline‐treated). Each biological replicate represents an individual mouse, and technical replicates (sequencing lanes from paired‐end libraries) were averaged prior to downstream analysis to avoid pseudoreplication. Raw read preprocessing involved adapter clipping and the filtering out of reads with length < 40 bp or mean *Q*‑score ≤30. After alignment to the Mus musculus genome (Ensembl version 88) and filtering of multi‐mapped reads, gene expression was quantified and normalised using the limma‐voom pipeline (limma package version 3.56.2, edgeR package version 3.42.4, R version 4.3.1). Specifically, raw count matrices were first normalised using the TMM (trimmed mean of *M*‐values) method implemented in the edgeR package, and then the voom function was applied to compute precision weights for linear modelling. The design matrix was constructed as model.matrix(∼0 + group), where group was a factor with levels ‘sham’ and ‘L‐OHP’. Gene‐level annotation was performed using the AnnotationDbi and Mus.musculus.ensembl.db packages. Differentially expressed genes (DEGs) between the two groups were identified with thresholds of |log_2_(Fold Change)| > 1 and adjusted *p* value < .05 (Benjamini–Hochberg false discovery rate correction) and visualised as a volcano plot. For palmitoylation‐related genes (including the ZDHHC family and other reported regulators), we extracted their normalised expression values, performed hierarchical clustering (ward.D2 linkage, Euclidean distance) with *z*‐score standardisation and generated a clustering heatmap to display their expression patterns across groups. Additionally, we selected genes significantly correlated with Zdhhc14 (|Pearson correlation coefficient| > .6, *p* < .05, calculated using the Pearson method in the stats package) or co‐expressed DEGs, conducted KEGG pathway enrichment analysis using the clusterProfiler package (version 4.8.1) with the enrichKEGG function, applying the Benjamini–Hochberg method for multiple‐testing correction with an adjusted *p*‐value (FDR) threshold of <.05, and visualised the enriched pathways as a bubble plot to elucidate biological processes associated with Zdhhc14. All statistical analyses were performed in R environment, and no technical replicates were treated as independent biological samples in any analysis. All raw sequencing data, processed expression matrices and detailed sample metadata are publicly accessible through the GEO accession GSE255097.

### 2.2 Animals

Male C57BL/6J (6–8 weeks old, 20–25 g) and Zdhhc14^−/−^ mice (on a C57BL/6J genetic background), both obtained from Cyagen Biosciences in Suzhou, were maintained in an SPF facility at 22 ± 2°C and 50–60% humidity on a 12‑h light‑dark cycle, with ad libitum chow and water. The Zdhhc14^−/−^ mice were generated on a C57BL/6J genetic background. Zdhhc14^+/−^ heterozygous mice were intercrossed to generate wild‐type (Zdhhc14^+/+^), heterozygous (Zdhhc14^+/−^) and knockout (Zdhhc14^−/−^) offspring. Genomic DNA was extracted from tail biopsies and genotyping was performed by PCR using primers specific for the targeting construct and the wild‐type allele, as validated by the vendor. All wild‐type control mice were age‐matched littermates of the Zdhhc14^−/−^ mice.

### 2.3 Animal grouping

Wild‑type mice were randomly assigned to experimental groups using a computer‐generated random number sequence. The allocation sequence was prepared by an investigator not involved in the experimental procedures and was kept in sealed opaque envelopes until after completion of all outcome measurements (allocation concealment). Wild‑type mice were randomised into groups of six (exceptions noted separately).

Saline group: Mice were given sterile saline via the i.p. route.

L‐OHP group (*n* = 24): L‐OHP was administered intraperitoneally to the mice. This group was further subdivided based on the time points post‐injection (Days 1, 3, 5 and 14), with six mice allocated to each time point.

L‐OHP + VX‐765 group: Mice were treated with VX‐765 following L‐OHP induction.

VX‐765 group: Mice received VX‐765 treatment alone without L‐OHP induction.

WT–AAV–shNC group: Mice in L‐OHP group were administered recombinant adeno‐associated virus serotype 9 (AAV9) carrying a negative control short hairpin RNA (shNC).

WT–AAV–shGbp5 group: Mice in L‐OHP group were administered AAV9 carrying shRNA targeting Gbp5 (shGbp5).

The Zdhhc14^−/−^ mice were divided into the following groups (*n* = 6).

Zdhhc14^−/−^ + L‐OHP group: L‑OHP was given intraperitoneally to the experimental mice.

Zdhhc14^−/−^ + saline group: Saline was injected i.p. into the mice.

Zdhhc14^−/−^ + AAV–NC group: Mice in L‐OHP group were injected with AAV9 carrying a negative control sequence for mouse Gbp5 overexpression.

Zdhhc14^−/−^ + AAV–Gbp5 group: Mice in L‐OHP group were injected with AAV9 carrying the mouse Gbp5 overexpression sequence.

### 2.4 AAV infection

AAV9 vectors expressing shGBP5 (AAV9–shGbp5), a scrambled control shRNA (AAV9–shNC), as well as AAV9 vectors overexpressing Gbp5 (AAV9–G5) and its corresponding negative control (AAV9–NC), were provided by Genechem Co., Ltd. (Shanghai, China). According to the experimental grouping, mice received an intramuscular injection of the corresponding AAV9 vectors (1 × 10^11^ vector genomes per mouse) into the gastrocnemius muscle 3 weeks prior to model induction. Successful transduction was confirmed by detecting GBP5 mRNA expression in the DRG using quantitative real‐time PCR (qPCR) at 3 weeks post‐injection.

### 2.5 OIPN mouse model

The OIPN mouse model was established as previously described.^24^ Following AAV infection, mice received intraperitoneal injections of L‐OHP (dissolved in.9% sterile saline immediately prior to administration; 3 mg/kg; Merck, Germany) for 5 days to construct the OIPN model. Control mice received.9% sterile saline at matching intervals and by the identical route. VX‐765 (100 mg/kg; MedChemExpress, Shanghai, China; prepared as a stock solution in 100% DMSO and further diluted with.9% sterile saline to a final DMSO concentration of ≤.5% v/v) was administered intraperitoneally for 5 consecutive days following each L‐OHP injection. All tissue processing and downstream molecular analyses (including haematoxylin–eosin (HE) staining, Western blotting, enzyme‐linked immunosorbent assay [ELISA] and immunofluorescence [IF]) were performed by investigators who were blinded to the treatment groups. Mice were euthanised at designated time points according to the experimental grouping. Sacrifice was achieved via cervical dislocation under 3% isoflurane anaesthesia. Blood from the orbital sinus was centrifuged at 3000×*g* over 15 min to yield plasma, which was kept at −80°C. L5 DRGs were collected, then either frozen in liquid nitrogen and held at −80°C or placed in 4% PFA for future examination.

### 2.6 Paw withdrawal threshold (PWT)evaluation

Mechanical sensitivity was assessed using the von Frey test to evaluate pain behaviour in OIPN mice. Mice were placed individually on a grid of elevated metal netting and acclimated for 30 min prior to testing. PWTs were determined by delivering von Frey filaments (.02–2.0 g) onto the hindpaw plantar aspect. The up‐down method was employed to determine the 50% withdrawal threshold. Tests were performed on Day 0 (baseline) and daily from Day 1 to Day 14. All behavioural assessments were conducted by an experimenter blinded to the treatment groups.

### 2.7 Cold sensitivity evaluation

Following completion of the von Frey testing, the left hindlimb received 100 µL of ice‑cold acetone on its plantar side, administered without forceful pressure. The duration (s) of nocifensive responses was recorded within a 1‐min observation window. The procedure was repeated twice, and the mean value of the two trials was calculated for each animal. Baseline cold sensitivity was established prior to the initiation of experimental treatments. An increase in the duration of nocifensive behaviour was taken as an indication of cold allodynia.

### 2.8 HE staining

To examine DRG morphological features, H&E staining was conducted with a commercial kit (Beyotime). Specimens were first immersed in 4% PFA, then paraffin‑embedded and sliced to 5‑µm thickness. After xylene deparaffinisation and rehydration via graded ethanol, sections were incubated with haematoxylin (5 min), rinsed, treated with 1% HCl‑alcohol and subsequently stained with eosin for 2 min. Following dehydration and clearing, coverslips were mounted and images were captured under a light microscope.

### 2.9 In vivo ubiquitination assay in mouse DRG

To validate K48‐linked ubiquitination of GBP5 in vivo, recombinant AAV9 vectors expressing HA–ubiquitin (HA–UB) wild‐type, K48R mutant or K63R mutant under the control of the CMV promoter were generated. Male C57BL/6J mice (6–8 weeks old) were randomly allocated into six groups (*n* = 3–4 per group): (1) AAV9–HA–UB–WT + vector control; (2) AAV9–HA–UB–WT + AAV9–Zdhhc14; (3) AAV9–HA–UB–K48R + vector control; (4) AAV9–HA–UB–K48R + AAV9–Zdhhc14; (5) AAV9–HA–UB–K63R + vector control; (6) AAV9–HA–UB–K63R + AAV9–Zdhhc14. AAV9 vectors (1 × 10^11^ vector genomes per mouse) were administered by intrathecal injection into the L5–L6 spinal cord segment in a volume of 10 µL PBS per mouse. Three weeks post‐injection, mice received intraperitoneal injections of MG132 (10 mg/kg) or vehicle 6 h prior to sacrifice to inhibit proteasomal degradation in vivo. DRG tissues (L4–L6) were rapidly dissected, homogenised in ice‐cold RIPA buffer containing protease and phosphatase inhibitors, and an aliquot was reserved as input. The remaining lysates were immunoprecipitated overnight at 4°C with anti‐GBP5 antibody, followed by capture with protein A/G magnetic beads for 2 h at 4°C. After washing, immune complexes were eluted and subjected to Western blotting. Ubiquitinated GBP5 was detected using anti‐ubiquitin or anti‐HA antibodies, and input and immunoprecipitation (IP) fractions were probed for GBP5, Zdhhc14 and β‐actin to verify expression and equal loading.

### 2.10 Isolation of primary neurons in DRG and cell culture

DRG neuron‐enriched cultures were isolated via enzymatic digestion and density gradient centrifugation.^25^ Briefly, L1–L6 DRGs were obtained from 5‑week‑old male mice. The DRGs were incubated in an enzyme solution containing 2.0 mg/mL collagenase and 50 U/mL dispase at 37°C for a designated duration. To remove myelin debris, the cell suspension underwent gradient centrifugation in 30% Percoll PLUS (GE Healthcare, Chicago, IL, USA). The neuronal sediment was re‑suspended in DMEM/F‑12 (Gibco) containing 10% FCS. Cells were then plated onto eight‐well chamber slides pre‐coated sequentially with 10 µg/mL poly‐L‐lysine and 10 µg/mL laminin. Purified neurons were incubated with.1 µg/mL L‑OHP for 48 h to generate an OIPN model in vitro. The oxaliplatin concentration (.1 µg/mL) and exposure duration (48 h) were selected based on dose–response and time‐course experiments using the Cell Counting Kit‐8 (CCK‐8) assay (Dojindo, Kumamoto, Japan). DRG neuron‐enriched cultures were seeded in 96‐well plates (1 × 10^4^ cells/well) and treated with oxaliplatin at concentrations ranging from 0 to 10 µg/mL for 24, 48 or 72 h. Cell viability was assessed by adding CCK‐8 reagent (10 µL/well) followed by incubation at 37°C for 2 h, and absorbance was measured at 450 nm. Each condition was tested in triplicate wells, and experiments were independently repeated three times (*n* = 3 biological replicates). As shown in Figure S1, treatment with.1 µg/mL oxaliplatin for 48 h reduced cell viability to approximately 55–60% of control. We therefore defined this condition as a sublethal concentration that induces moderate cellular injury without causing massive cell death, rather than being strictly non‐cytotoxic. This condition was used to model the partial neuronal injury characteristic of OIPN while preserving sufficient viable cells for mechanistic analyses.

HEK293T cells (SUNNCELL, Wuhan, China) were cultured in DMEM (Gibco) supplemented with 10% FBS.

### 2.11 Cell transfection

Log‑phase DRG neurons and HEK293T cells were plated into six‑well dishes and subsequently subjected to transfection with short hairpin RNA targeted Zdhhc14 (shZdhhc14), shRNA negative control (shNC), pcDNA3.1–GBP5 and its negative control (pcDNA3.1) (Tsingke, Beijing, China). Transient transfection was performed using Lipofectamine 3000 (Invitrogen, Carlsbad, CA, USA). After 48‑h transfection, cells were pelleted for later use.

### 2.12 qPCR

Total RNA was extracted using Trizol reagent (Invitrogen). After cDNA preparation with a Takara kit (Tokyo, Japan), the products were quantified by real‑time PCR with Vazyme's SYBR Green reaction mixture. To determine relative expression, we normalised all mRNA data to β‑actin (internal control) and then applied the 2^−ΔΔCt^ formula to compute the fold changes. Primer oligonucleotides are listed below: Zdhhc14, 5′‐CTCGCCTCCTGTGTGACAAT‐3′ (sense) and 5′‐GGCTGTTTTGAATTATTCCTTACCA‐3′ (antisense); Zdhhc1, 5′‐ATCCTTCTGGGCCTGCTTTC‐3′ (sense) and 5′‐GGGCATGACTCCAGCTCC‐3′ (antisense); Zdhhc9, 5′‐CGTGAGGAGCGTTCCATTCC‐3′ (sense) and 5′‐AGAGCCCTGAACAAGGGACA‐3′ (antisense); Zdhhc20, 5′‐CGGCAAGAGACCTGCCTATC‐3′ (sense) and 5′‐TCTCTGTAGATTTCCTGCGGC‐3′ (antisense); Zdhhc18, 5′‐ATCAGAAGTGATGACCCGGC‐3′ (sense) and 5′‐ACCTAGGTTTGGGGAGCAGA‐3′ (antisense); Zdhhc15, 5′‐TGCTAGGCAACCACCTTCTG‐3′ (sense) and 5′‐CCCAGACAGAGCCATCTTCC‐3′ (antisense); β‐actin, 5′‐CAGCCTTCCTTCTTGGGTATG‐3′ (sense) and 5′‐GGCATAGAGGTCTTTACGGATG‐3′ (antisense).

### 2.13 Western blot

RIPA (Beyotime) supplemented with inhibitors was used to lyse the three cell types (DRGs, primary neurons, HEK293T). Protein concentration was determined by BCA, and 30‑µg aliquots underwent 10% SDS‑PAGE, PVDF transfer, 1‑h blocking with 5% milk in TBST and overnight primary antibody incubation at 4°C. Afterwards, HRP‐conjugated secondary antibodies (1:10 000, ab6721; Abcam, Cambridge, MA, USA) were applied for 1 h at room temperature. Protein bands were visualised using enhanced chemiluminescence reagent (Beyotime). The primary antibodies are as follows: anti‐NLRP3 (1:1000, ab263899; Abcam), anti‐GSDMD‐N (1:1000, ab215203; Abcam), anti‐CL‐CASP1 (1:1000, #4199; Cell Signaling, Danvers, MA, USA), anti‐Zdhhc14 (1:1000, PA5‐49367; Thermo fisher Scientific), anti‐GBP5 (1:1000, ab313390; Abcam), anti‐palmitoylation (1:1000, P49299; Abmart, Shanghai, China), anti‐ubiquitination (1:1000, 10201‐2‐AP; Proteintech), anti‐β‐actin (1:5000, ab6276; Abcam), anti‐Myc (1:1000, ab32072; Abcam), anti‐HA (1:1000, ab236632; Abcam), anti‐Flag (1:1000, ab205606; Abcam).

### 2.14 Enzyme‐linked immunosorbent assay

Plasma and DRG samples from mice, together with DRG neuron‐enriched cultures, were assayed for IL‑1β, IL‑6, TNF‑α and IL‑18 using respective commercial ELISA kits (all supplied by Beyotime). The entire procedure adhered to the provider's protocol, and the resulting optical densities were read at 450 nm on a microplate reader.

### 2.15 IF staining

IF staining for Zdhhc14, NeuN, GFAP and IBA1 in mouse DRG tissues and for Zdhhc14 and GBP5 in HEK‐293T cells was conducted using an IF Staining Kit (Beyotime). Mouse DRG frozen sections, after fixation/permeabilisation/blocking, were incubated at 4°C overnight with anti‑Zdhhc14, ‑NeuN, ‑GFAP and ‑IBA1 primary antibodies, then with fluorescent secondaries at RT in darkness. As the DRG is a peripheral nervous system structure, GFAP‐positive cells herein refer to satellite glial cells (SGCs) rather than astrocytes, and IBA1‐positive cells refer to resident or infiltrating macrophages rather than microglia. For HEK293T cells grown on coverslips, the same fixation, permeabilisation and blocking steps were performed, and the cells were next incubated with anti‑Zdhhc14 and anti‑GBP5 primary antibodies at 4°C overnight and the corresponding fluorescent secondary antibodies thereafter. After DAPI staining, samples were mounted and viewed by fluorescence microscopy.

### 2.16 Lactate dehydrogenase release assay

To evaluate cell damage, we applied the lactate dehydrogenase (LDH) assay kit (Beyotime) to DRG neuron‐enriched cultures. Neurons were seeded overnight in 96‑well plates (1 × 10^4^ per well). Following transfection, the plates were subjected to centrifugation (400×*g*, 5 min). Afterwards, 120 µL of the supernatant from each well was collected into another 96‑well dish. Freshly prepared LDH detection working solution (60 µL per well), consisting of substrate mix, INT solution (1×) and enzyme solution at a 1:1:1 ratio, was added to each well. After a 30‐min dark incubation at room temperature, the plate was measured for absorbance at 490 nm via a microplate reader. For maximum LDH release control, neurons were treated with LDH release reagent (provided in the kit) at 1 h prior to supernatant collection. Background control wells containing medium only were included.

### 2.17 Flow cytometry

Cultured primary neurons were first harvested, rinsed twice in ice‐cold PBS and re‐dispersed in the culture medium to a proper cell density. Pyroptosis was assessed using the FAM‐FLICA® Caspase‐1 (YVAD) assay kit (ImmunoChemistry Technologies, Bloomington, MN, USA), which detects active caspase‐1 together with membrane permeability. In brief, the cell suspension was incubated with the FAM‐YVAD‐FMK FLICA reagent (1× in culture medium) for 60 min at 37°C under 5% CO_2_ in the dark, allowing the cell‐permeable FAM‐YVAD‐FMK probe to covalently bind active caspase‐1 within intact cells. The cells were then washed twice with the kit's wash buffer (Apoptosis Wash Buffer) to remove unbound reagent and free FAM‐YVAD‐FMK, and subsequently stained with propidium iodide (PI; provided in the kit) for 5 min at room temperature in the dark to identify membrane‐compromised cells. Flow cytometry was then promptly performed. The pyroptotic cell fraction was defined and quantified as the FAM‐FLICA^+^/PI^+^ double‐positive population – that is, cells simultaneously carrying active caspase‐1 and a permeabilised plasma membrane – whereas FAM‐FLICA^+^/PI^−^ cells (active caspase‐1 without membrane permeability) were not counted as pyroptotic.

### 2.18 Immunoprecipitation–mass spectrometry

HEK293T cells transfected with Flag‐tagged Zdhhc14 were lysed in IP buffer with protease inhibitors. Lysates were incubated with anti‐Flag antibody overnight at 4°C, captured on Protein A/G magnetic beads, washed and eluted. Bound proteins were digested with trypsin (37°C, 16 h), and peptides were desalted using C18 SpinTips.

Peptides were analysed on an EASY‐nLC 1200 system coupled to a Q Exactive HF‐X mass spectrometer (Thermo Fisher Scientific). Mobile phase A was.1% formic acid in water; mobile phase B was.1% formic acid in acetonitrile. Peptides were separated with a linear gradient from 5 to 38% B over 52 min at a flow rate of 300 nL/min. Full MS scans (350–1500 *m*/*z*) were acquired at 60 000 resolution and MS/MS scans at 15 000 resolution in data‐dependent acquisition mode.

Raw data were processed with Proteome Discoverer (v2.4.0) against the UniProt human database. Trypsin was set as the digestion enzyme with a maximum of 2 missed cleavages. Cysteine carbamidomethylation was the fixed modification; methionine oxidation and protein N‐terminal acetylation were variable modifications. Three biological replicates were performed.

### 2.19 Co‐immunoprecipitation (Co‐IP)

To probe the Zdhhc14–GBP5 association, we employed a commercial immunoprecipitation kit (Beyotime). HEK293T cells received co‑transfection with Flag‑tagged Zdhhc14 and Myc‑tagged GBP5 constructs. At 48 h post‑transfection, lysis was carried out in IP buffer supplemented with protease blockers. The cleared lysates were then reacted overnight at 4°C with anti‑Flag, anti‑Myc or control IgG. Following this, protein A/G agarose was added and left for another 3 h at the same temperature. After thorough washing, the captured material was released and immunoblotted with antibodies recognising Zdhhc14, GBP5, Flag and Myc to confirm the specific binding. To validate the key interface residues mediating the Zdhhc14–GBP5 interaction, site‐directed mutagenesis was performed. Point mutants of Zdhhc14 (L378A and T423A) and GBP5 (R499A, R515A and Q437A) were generated using a site‐directed mutagenesis kit (Takara) according to the manufacturer's protocol, and all mutations were confirmed by DNA sequencing. HEK293T cells were co‐transfected with Flag‐tagged Zdhhc14 wild‐type or point mutants (L378A, T423A) together with Myc‐tagged GBP5 wild‐type or with Myc‐tagged GBP5 wild‐type or point mutants (R499A, R515A, Q437A) together with Flag‐tagged Zdhhc14 wild‐type. Co‐IP assays were performed as described above, and the effects of these mutations on protein–protein interaction were evaluated by Western blotting.

### 2.20 Immunoprecipitation

To examine GBP5 palmitoylation, HEK293T lysates (prepared in IP buffer with protease inhibitors) were incubated overnight at 4°C with anti‑GBP5, then captured on Protein A/G beads for 2 h. After washes, bound proteins were eluted and blotted with anti‑palmitoyl antibody, using the Co‑IP Kit (Thermo) as per the manufacturer's instructions.

To map the critical binding domains between Zdhhc14 and GBP5, truncated expression plasmids of Zdhhc14 (N‐terminal, DHHC catalytic, C‐terminal domains) and GBP5 (GTPase, middle, C‐terminal helical domains) were generated. Flag‐tagged Zdhhc14 (full‐length or truncated) and HA‐tagged GBP5 (full‐length or truncated) were co‐transfected into HEK293T cells. Lysis and anti‑Flag bead‑based immunoprecipitation were performed on the cells at 48 h after transfection. Co‐precipitated proteins were detected by Western blot to identify the key binding domains, with input samples confirming equal expression of full‐length and truncated constructs.

### 2.21 Acyl‐biotin exchange assay

GBP5 palmitoylation was evaluated using the acyl‐biotin exchange (ABE) method. Lysates were gently rotated with NEM (4°C, 1 h). Anti‑GBP5‐coated magnetic beads were then applied to immunoprecipitate GBP5. Following washes, bead‑associated proteins were incubated with 1 M hydroxylamine (HAM, pH 7.2) for 1 h at RT to cleave palmitoyl thioesters, then labelled with biotin‑BMCC (5 µM, 2 h at 4°C). Finally, the eluates were recovered in SDS sample buffer, denatured by heating (100°C, 10 min) and analysed via immunoblotting.

### 2.22 Protein stability assay

To assess GBP5 stability, HEK293T cells received 10 µM CHX (MedChemExpress) to inhibit translation, and GBP5 levels were quantified via Western blot at 0, 2, 4 and 8 h post‑treatment.

### 2.23 Ubiquitination assay

To investigate GBP5 ubiquitylation in HEK‑293T cells, co‑transfections were performed with plasmids expressing Zdhhc14, HA‑ubiquitin or its K48R/K63R mutants as designated. After 24 h, 10 µM MG132 was administered for 6 h to inhibit proteasomal degradation. Cells were then lysed in chilled RIPA buffer containing protease/phosphatase inhibitors; an aliquot was reserved as input, while the rest was immunoprecipitated overnight with anti‑GBP5 and then captured by protein A/G magnetic beads (2 h, 4°C). Following washes, the immune complexes were eluted and subjected to Western blotting. Ubiquitylated GBP5 was revealed using anti‑ubiquitin (or anti‑HA), and both input and IP fractions were probed for GBP5 and Zdhhc14 with corresponding antibodies to verify expression.

### 2.24 Molecular dynamics simulation analysis

The optimal docking conformation of the Zdhhc14–GBP5 complex was subjected to molecular dynamics (MD) simulation using the GROMACS package. The system was solvated in a water box, neutralised with counterions and minimised before equilibration under NVT and NPT ensembles. A 100‐ns production run was subsequently performed. To evaluate the structural stability and local flexibility of the complex, the root mean square deviation (RMSD) and root mean square fluctuation were calculated across the trajectory. Meanwhile, the number of intermolecular hydrogen bonds was monitored to assess the persistence of the interaction. Furthermore, the molecular mechanics Poisson–Boltzmann surface area (MM‐PBSA) method was employed for per‐residue binding free energy decomposition. Key residues with substantial energy contributions (including GBP5–ARG515, GBP5–ARG499, Zdhhc14–THR423, GBP5–GLN437 and Zdhhc14–LEU378) were identified based on the energy decomposition results and selected for subsequent site‐directed mutagenesis experiments to validate their potential importance in the interaction.

### 2.25 Structural modelling and in silico analyses

The three‐dimensional structure of mouse GBP5 was predicted using AlphaFold3 via the AlphaFold Server with default settings. Five independent models were generated per prediction, and the top‐ranked model was used for structural analyses unless otherwise indicated. Model confidence was evaluated using the mean predicted local distance difference test (pLDDT) score, the predicted template modelling (pTM) score, the predicted aligned error (PAE) matrix and multiple‐sequence‐alignment (MSA) coverage. To model C12‐palmitoylated GBP5, S‐palmitoylation was introduced at Cys12 through the CCD_P1L covalent modification in the AlphaFold Server input, and five independent models were generated. Structures were visualised and analysed in PyMOL. The relative solvent‐accessible surface area (SASA) of cysteine residues was calculated in PyMOL and normalised to the theoretical maximum accessibility of a cysteine residue in a Gly–Cys–Gly reference tripeptide. The membrane‐interacting region of GBP5 was annotated based on UniProt. Evolutionary conservation analysis was performed using the ConSurf web server (https://consurf.tau.ac.il). Computational prediction of cysteine post‐translational modifications (S‐palmitoylation, S‐nitrosylation, S‐glutathionylation, S‐sulfenylation, S‐sulfinylation and S‐sulfhydration) was performed with default thresholds. For the comparison of lysine accessibility between unmodified and C12‐palmitoylated GBP5, the SASA of all lysine residues was computed for each of the five models per condition using the Shrake–Rupley algorithm (probe radius 1.4 Å); per‐residue values were compared between conditions using Welch's *t*‐test with Benjamini–Hochberg correction for multiple comparisons. Structural superposition was performed on the GTPase domain (residues 35–277), and Cα RMSD was calculated in PyMOL.

### 2.26 Statistical analysis

Statistical analysis was performed using GraphPad Prism 8.0 (GraphPad Software, San Diego, CA, USA) or SPSS software. For longitudinal behavioural experiments (paw withdrawal threshold and cold sensitivity), data were analysed using two‐way repeated‐measures ANOVA with treatment and time as factors, followed by Bonferroni post‐hoc tests. For comparisons at individual time points or between two groups, unpaired Student's *t*‐test was used; for multiple group comparisons of independent samples, one‐way ANOVA with Tukey's post‐hoc test was applied. For animal experiments, *n* = 6 represents biological replicates (individual mice) per group; for in vitro experiments, each assay was independently repeated at least three times as biological replicates. For each biological replicate, a minimum of three technical repeats were included, and all values are given as means ± SD. The experimental unit for behavioural analyses was the individual mouse. Data normality and variance homogeneity were checked via Shapiro–Wilk and Levene's tests, respectively. Significance was set at *p* < .05. Sample size for animal experiments (*n* = 6 per group) was determined based on our previous experience with this OIPN model and pilot study variance estimates, which indicated that this sample size provides sufficient power (>80%) to detect biologically meaningful differences in behavioural and molecular outcomes.

## 3 RESULTS

### 3.1 Zdhhc14 is up‐regulated in the DRG of L‐OHP‐treated mice

To explore the molecular basis of L‑OHP‑evoked peripheral neuropathy, we analysed DEGs between L‐OHP‐treated and sham groups in the GSE255097 dataset. A volcano plot displays the findings (Figure 1A). Clustering analysis of palmitoylation‐related genes revealed that Zdhhc16, Zdhhc14 and Zdhhc1, among others, were significantly differentially expressed between the L‐OHP and sham groups (Figure 1B). The expression patterns of these genes are clearly illustrated in a bar graph (Figure 1C). Next, we constructed a L‐OHP‐induced OIPN mouse model, and the workflow of model construction is shown in Figure 1D. C57BL/6J mice were subjected to consecutive intraperitoneal injections of L‐OHP for 5 days, and sacrificed on Day 14 after the initial injection for subsequent analyses. Relative to the saline cohort, the PWT in the L‐OHP group began to significantly decrease from Day 2 post‐modelling and persisted until Day 14, indicating successful induction of mechanical hyperalgesia by L‐OHP (Figure 1E). The cold sensitivity test demonstrated that L‐OHP also significantly induced cold allodynia in mice (Figure 1F). HE staining revealed progressive pathological damage in the DRG tissues of L‐OHP‐treated mice, characterised by abnormal cellular morphology and disorganised structure, with escalating injury severity over time (Figure 1G). Subsequently, we validated the expression of differentially expressed Zdhhc family genes from the GSE255097 dataset in the DRG of L‐OHP‐treated mice. The results revealed that only Zdhhc14 expression was significantly elevated as early as Day 1 following L‐OHP induction and continued to up‐regulate over time (Figure 1H). Zdhhc1 expression was significantly up‐regulated on Day 5 post‐induction (Figure 1I), whereas Zdhhc9 and Zdhhc20 showed no significant changes throughout the induction period (Figure 1J,K). In contrast, Zdhhc18 and Zdhhc15 were down‐regulated starting on Days 5 and 3, respectively (Figure 1L,M). These findings indicate that Zdhhc14 expression is the most sensitive to L‐OHP induction. Consequently, we further examined Zdhhc14 protein levels in the DRG and observed a trend consistent with its mRNA expression (Figure 1N). IF staining was performed to co‐localise Zdhhc14 with the cell‐specific markers NeuN (neurons), GFAP (satellite glial cells) and IBA1 (DRG macrophages). The results revealed extensive co‐localisation of Zdhhc14 with NeuN, confirming that Zdhhc14 is predominantly expressed in neurons (Figure 1O). Subsequently, primary neurons were isolated from mouse DRG, and Zdhhc14 expression was detected. The mRNA, protein levels, as well as expression intensity, were significantly elevated in these primary neurons (Figure 1P–R), further confirming that L‐OHP induces Zdhhc14 up‐regulation. These findings identify Zdhhc14 as a potential key regulatory factor in L‐OHP‐induced OIPN and highlight its predominant expression in the DRG.

**FIGURE 1:**
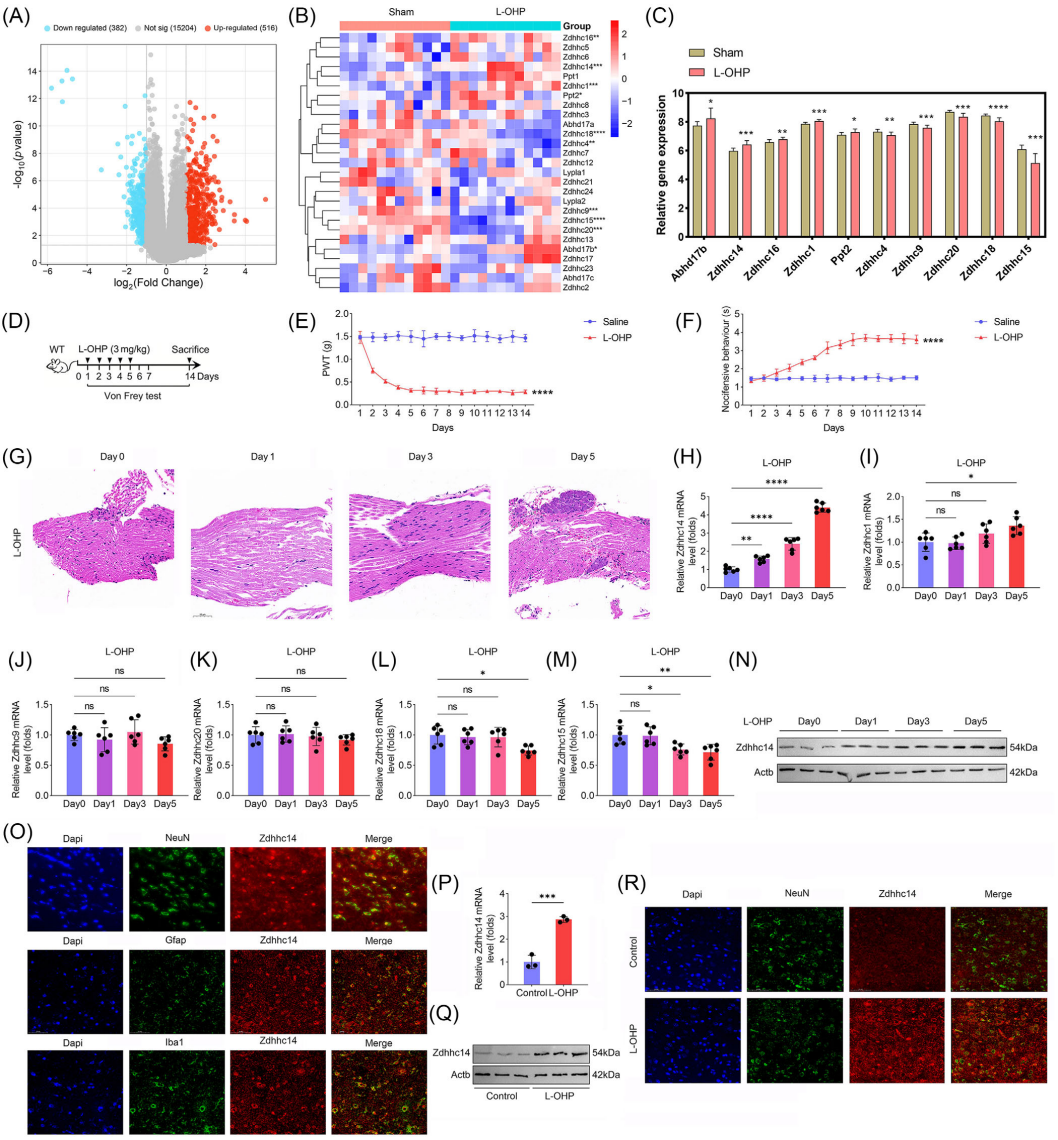
Zdhhc14 is up‐regulated in the neuron of L‐OHP‐treated mice. (A) The volcano plot showed the DEGs between L‐OHP‐treated and sham groups in the publicly available GSE255097 dataset (https://www.ncbi.nlm.nih.gov/geo/query/acc.cgi?acc=GSE255097). (B) Clustering heatmap of palmitoylation‐related genes. (C) Bar graph showing expression patterns of differentially expressed Zdhhc family genes. (D) Schematic workflow of OIPN mouse model construction. (E) PWT of mice over 14 days. Two‐way repeated‐measures ANOVA with Bonferroni post‐hoc test; *n* = 6 mice per group (biological replicates). (F) Evaluation of cold allodynia in mice over the 14‐day observation period. Two‐way repeated‐measures ANOVA with Bonferroni post‐hoc test; *n* = 6 mice per group (biological replicates). Data are means ± SD. (G) HE staining of DRG tissues showing histopathological changes at different time points. (H‐M) mRNA expression levels of Zdhhc14, Zdhhc1, Zdhhc9, Zdhhc20, Zdhhc18 and Zdhhc15 in DRG tissues were detected by qPCR. (N) Protein levels of Zdhhc14 in DRG tissues were detected by Western blot. (O) IF staining showing co‐localisation of Zdhhc14 with NeuN (neurons), GFAP (satellite glial cells) and IBA1 (DRG macrophages) in DRG tissues. (P) mRNA expression levels of Zdhhc14 in DRG neuron‐enriched cultures were detected by qPCR. (Q) Protein levels of Zdhhc14 in DRG neuron‐enriched cultures were detected by Western blot. (R) The expression intensity of Zdhhc14 in primary DRGs was evaluated by IF staining. ns, no significance, *p < .05, **p < .01, ***p < .001 and ****p < .0001.

### 3.2 Pyroptosis is increased in mice with L‐OHP‐induced OIPN

Using the GSE255097 dataset, we identified Zdhhc14‑correlated genes and performed KEGG pathway enrichment analysis, which showed pyroptosis pathway overrepresentation (Figure 2A). To investigate whether pyroptosis occurs in the L‐OHP‐induced OIPN mouse model, we treated mice with the pyroptosis inhibitor VX‐765. We observed that PWT in both the VX‐765 and saline groups remained at baseline levels, indicating that VX‐765 alone does not affect PWT. In contrast, the L‐OHP + VX‐765 group exhibited a significant recovery in PWT, suggesting that the pyroptosis inhibitor VX‐765 effectively alleviates L‐OHP‐induced mechanical hyperalgesia (Figure 2B). Similarly, cold sensitivity assessment revealed that L‐OHP‐induced cold allodynia was markedly attenuated by VX‐765 co‐treatment, with the L‐OHP + VX‐765 group showing significantly reduced nocifensive duration compared with the L‐OHP group, while VX‐765 alone had no effect on baseline cold sensitivity (Figure 2C). Relative to saline‐treated controls, L‑OHP treatment yielded significantly higher concentrations of IL‑18, IL‑1β, IL‑6 and TNF‑α in plasma and DRG tissues. Of note, VX‑765 markedly lowered the levels of the above pro‑inflammatory cytokines in OIPN mice relative to the L‑OHP cohort. Similarly, VX‐765 also decreased cytokine expression in normal mice. These results indicate that the pyroptosis inhibitor VX‐765 significantly suppresses L‐OHP‐induced inflammation (Figure 2D–K). Moreover, L‐OHP administration up‐regulated pyroptosis‐related core proteins (NLRP3, GSDMD‐N and CL‐CASP1) in DRG tissues compared with saline controls. However, these protein levels were markedly decreased in the L‐OHP + VX‐765 group relative to the L‐OHP group (Figure 2L), confirming VX‐765‐mediated suppression of pyroptosis in L‐OHP‐induced OIPN mice. Collectively, these results demonstrate that L‐OHP enhanced pyroptotic activity in the OIPN mouse model.

**FIGURE 2:**
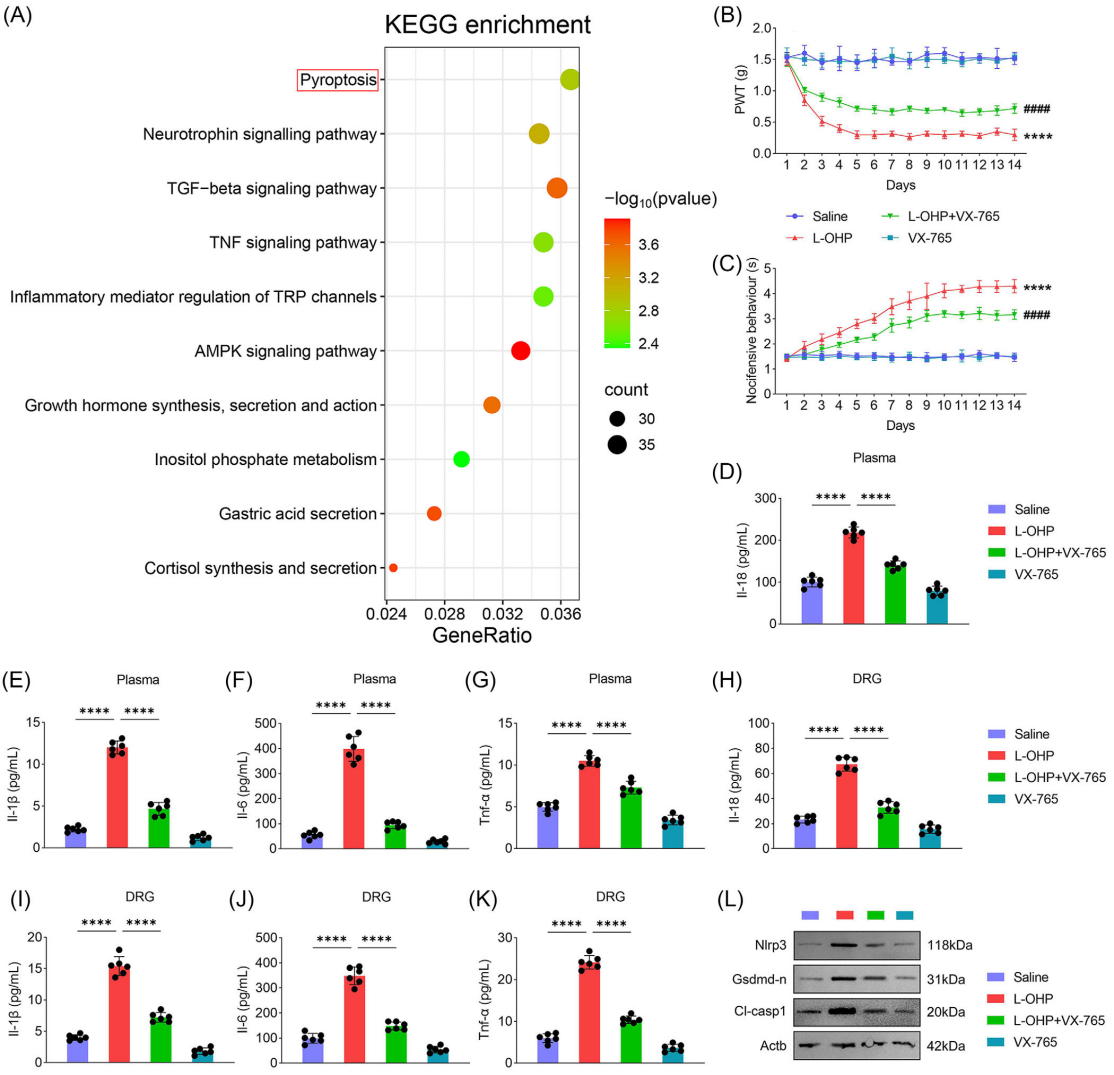
Pyroptosis is increased in mice with L‐OHP‐induced OIPN. (A) KEGG pathway enrichment analysis of genes correlated with Zdhhc14 in the publicly available GSE255097 dataset (https://www.ncbi.nlm.nih.gov/geo/query/acc.cgi?acc=GSE255097). (B and C) PWT and cold allodynia mice in saline, VX‐765, L‐OHP and L‐OHP + VX‐765 groups from Day 0 to Day 14. Two‐way repeated‐measures ANOVA with Bonferroni post‐hoc test; *n* = 6 mice per group (biological replicates). Data are means ± SD. (D–G) Plasma levels of IL‐18, IL‐1β, IL‐6 and TNF‐α detected by ELISA. (H–K) DRG tissue levels of IL‐18, IL‐1β, IL‐6 and TNF‐α detected by ELISA. (L) Western blot images of pyroptosis‐related proteins (NLRP3, GSDMD‐N and CL‐CASP1) in DRG tissues. ****p < .0001.

### 3.3 Zdhhc14 deletion suppresses L‐OHP‐induced OIPN phenotypes and pyroptosis

To explore Zdhhc14's role in L‑OHP‑evoked OIPN and pyroptosis modulation, we generated Zdhhc14‐specific knockout mice. The absence of Zdhhc14 protein in the DRG of Zdhhc14^−/−^ mice was observed (Figure 3A). Compared with the WT + L‐OHP group, the Zdhhc14^−/− ^+ L‐OHP group exhibited significantly higher PWT, indicating that Zdhhc14 deletion markedly alleviates L‐OHP‐induced mechanical hyperalgesia (Figure 3B). Similarly, cold sensitivity assessment revealed that L‐OHP‐induced cold allodynia was significantly attenuated in Zdhhc14^−/−^ mice, with the Zdhhc14^−/−^ + L‐OHP group showing markedly reduced nocifensive duration compared with the WT + L‐OHP group, whereas Zdhhc14 deletion alone had no effect on baseline cold sensitivity (Figure 3C). HE staining further revealed reduced DRG tissue damage in Zdhhc14^−/−^ + L‐OHP mice relative to WT + L‐OHP mice (Figure 3D). Relative to the WT + L‑OHP group, the Zdhhc14^−^/^−^ + L‑OHP group showed markedly reduced plasma and DRG levels of IL‑18, IL‑1β, IL‑6 and TNF‑α (Figure 3E–L). Finally, Western blot analysis revealed that NLRP3, GSDMD‐N and CL‐CASP1 protein expression in DRG tissues were markedly decreased in the Zdhhc14^−/−^ + L‐OHP group compared with the WT + L‐OHP group (Figure 3M), indicating suppressed pyroptosis. Notably, no significant differences were observed between the Zdhhc14^−/−^ + saline and WT + saline groups, suggesting that Zdhhc14 functions primarily under L‐OHP‐induced OIPN conditions. Collectively, these results indicate that Zdhhc14 deletion significantly attenuates L‐OHP‐induced cold allodynia, neuropathic pain, inflammation and pyroptosis in mice.

**FIGURE 3:**
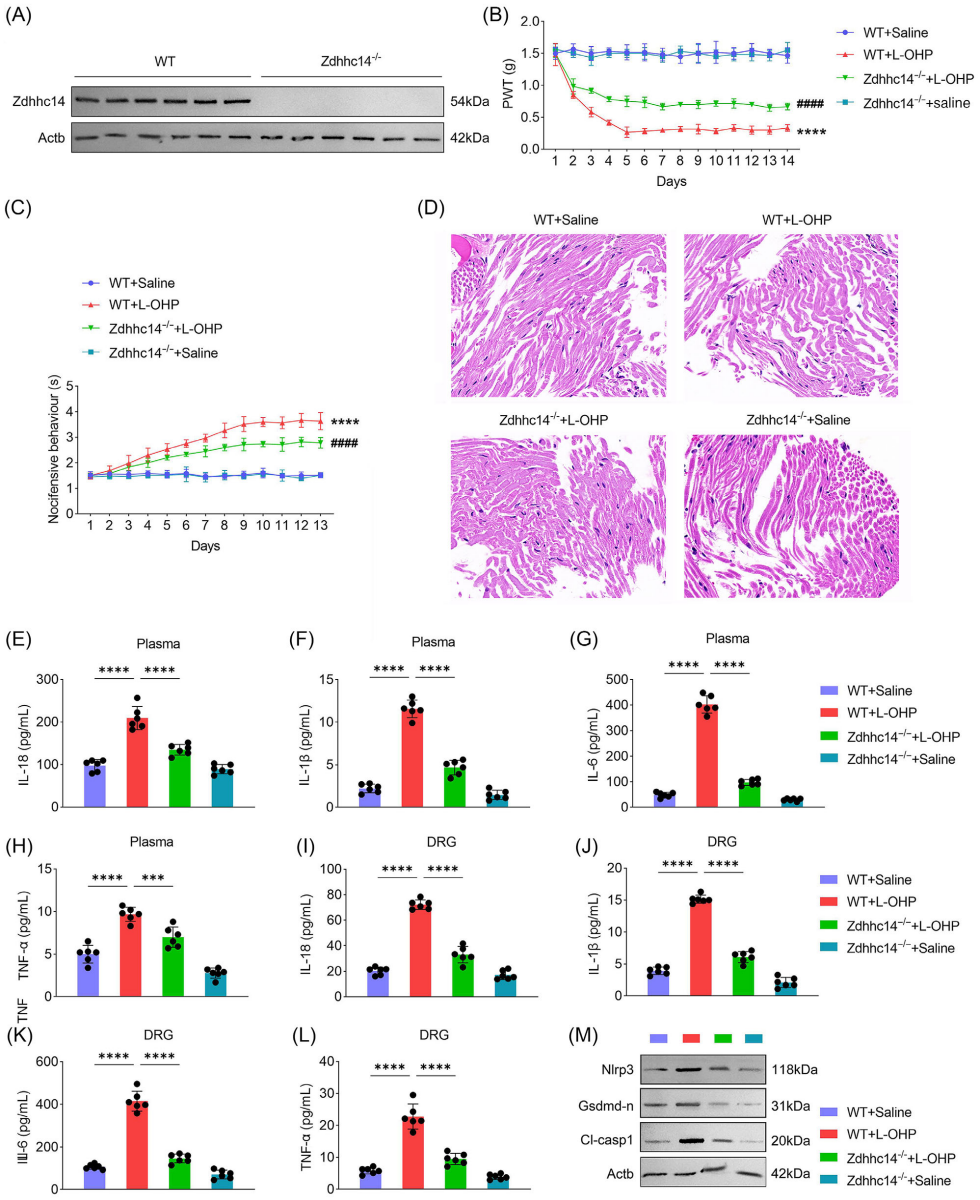
Zdhhc14 deletion suppresses L‐OHP‐induced OIPN phenotypes and pyroptosis. (A) The protein level of Zdhhc14 in DRG tissues of WT and Zdhhc14^−/−^ mice detected by Western blot. (B and C) PWT and cold allodynia of mice in WT + saline, WT + L‐OHP, Zdhhc14^−/−^ + saline and Zdhhc14^−/−^ + L‐OHP groups from Day 0 to Day 14. Two‐way repeated‐measures ANOVA with Bonferroni post‐hoc test; *n* = 6 mice per group (biological replicates). Data are means ± SD. (D) HE staining of DRG tissues was performed to detect histopathological changes. (E–H) IL‐18, IL‐1β, IL‐6 and TNF‐α levels in plasma detected by ELISA. (I–L) IL‐18, IL‐1β, IL‐6 and TNF‐α levels in DRG tissues detected by ELISA. (M) The protein expression levels of NLRP3, GSDMD‐N and CL‐CASP1 in DRG tissues detected by Western blot. ****p < .0001.

### 3.4 Zdhhc14 knockdown inhibits pyroptosis in L‐OHP‐induced DRG neuron‐enriched cultures

Furthermore, we validated the functional role of Zdhhc14 in L‐OHP‐induced pyroptosis in DRG neuron‐enriched cultures. Zdhhc14 knockdown significantly reduced Zdhhc14 protein levels in cultured cells (Figure 4A). The LDH release rate was markedly elevated in the L‐OHP group. Notably, the L‑OHP + shZdhhc14 group showed a statistically significant reduction in LDH release, suggesting that Zdhhc14 knockdown alleviates L‐OHP‐induced cytotoxicity (Figure 4B). Additionally, ELISA revealed that L‐OHP significantly elevated the concentrations of IL‐18 and IL‐1β, whereas these were markedly decreased following Zdhhc14 knockdown (Figure 4C,D). Flow cytometric data revealed a marked rise in pyroptosis for L‑OHP‑treated cells versus control. Moreover, Zdhhc14 knockdown markedly decreased the pyroptosis rate (Figure 4E,F). Western blot analysis further confirmed that L‐OHP treatment up‐regulated pyroptosis‐related core proteins (NLRP3, GSDMD‐N and CL‐CASP1), whereas these protein levels were down‐regulated following Zdhhc14 knockdown (Figure 4G). Collectively, these results demonstrate that Zdhhc14 knockdown significantly suppresses L‐OHP‐induced cell damage, inflammatory cytokine release and pyroptosis in DRG neuron‐enriched cultures.

**FIGURE 4:**
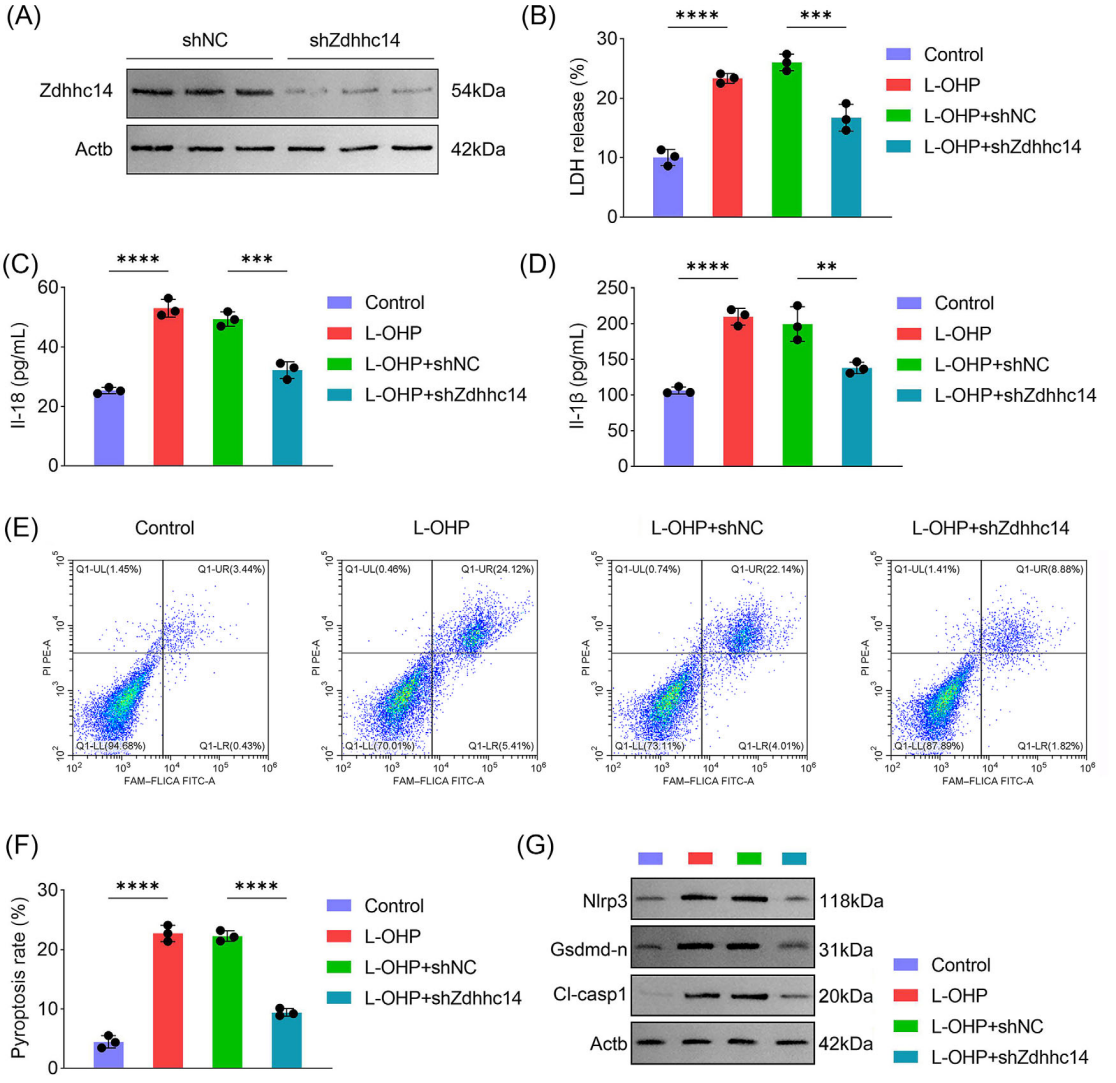
Zdhhc14 knockdown inhibits pyroptosis in L‐OHP‐induced DRG neuron‐enriched cultures. (A) The protein levels of Zdhhc14 in DRG neuron‐enriched cultures after knockdown detected by Western blot. (B) LDH release rate of DRG neuron‐enriched cultures. (C and D) IL‐18 and IL‐1β levels in DRG neuron‐enriched cultures detected by ELISA. (E) Representative flow cytometry plots and (F) quantitative analysis of pyroptosis rate. (G) Protein expression levels of NLRP3, GSDMD‐N and CL‐CASP1 in DRG neuron‐enriched cultures detected by Western blot. *p < .01, ***p < .001 and ****p < .0001.

### 3.5 ZDHHC14 enhances GBP5 protein stability through Cys12‐dependent modification and by suppressing K48‐linked ubiquitination degradation

Our previous immunoprecipitation–mass spectrometry (IP‐MS) analysis revealed that GBP5 exhibited the highest enrichment over control among ZDHHC14‐interacting proteins (Table S1 and Figure S2). To validate this, Co‐IP experiments in HEK‐293T cells confirmed the physical association between ZDHHC14 and GBP5 (Figure 5A,B). ZDHHC14 overexpression significantly increased both GBP5 palmitoylation level and GBP5 protein expression (Figure 5C). Notably, the protein and palmitoylation levels of GBP5 were not affected by overexpression of ZDHHC1 or ZDHHC16 (Figure S3A,B). IP–ABE assays further confirmed that ZDHHC14 is associated with increased palmitoylation of GBP5, suggesting that ZDHHC14, as a palmitoyltransferase, facilitates GBP5 palmitoylation in this context (Figure 5D,E). IF staining further demonstrated co‐localisation of ZDHHC14 and GBP5 in cells (Figure 5F). CHX chase experiments revealed that GBP5 protein degradation was significantly slower in the ZDHHC14‐overexpressing group, indicating that ZDHHC14 enhances GBP5 protein stability (Figure 5G). Ubiquitination assays showed that ZDHHC14 overexpression markedly reduced GBP5 ubiquitination levels, suggesting that ZDHHC14 stabilises GBP5 by inhibiting ubiquitin‐mediated degradation (Figure 5H). Notably, ubiquitination site mutation experiments revealed that GBP5 ubiquitination in the K48R mutant group was unaffected by ZDHHC14 overexpression (Figure 5I), confirming that ZDHHC14 suppresses GBP5 degradation via K48‐linked ubiquitin chains. Ubiquitination assays in mouse DRG tissues also confirmed the same results (Figure S4). Additionally, we predicted three potential palmitoylation sites (C12, C578 and C579) on GBP5 and generated cysteine‐to‐serine (C to S) mutants. Results showed that only the C12S mutation reduced GBP5 palmitoylation and protein levels in cells with ZDHHC14 overexpression (Figure 5J). To directly test whether the residue at position 12 mechanistically links to the suppression of K48‐linked ubiquitination and protein stabilisation, we directly compared GBP5–WT and GBP5–C12S. As shown in Figure S5, in the presence of ZDHHC14 overexpression, GBP5–WT exhibited markedly reduced total ubiquitination and K48‐linked ubiquitination, together with a prolonged protein half‐life; by contrast, the C12S mutation abolished these effects of ZDHHC14, as evidenced by persistently high levels of ubiquitination and K48‐linked ubiquitination (Figure S5A) and a significantly shortened half‐life in CHX chase assays (Figure S5C), accompanied by lower steady‐state protein abundance (Figure S5B). Quantitative analysis confirmed that the stabilising effect of ZDHHC14 was lost in the C12S mutant (Figure S5C). These results demonstrate that Cys12 is required for ZDHHC14 to suppress K48‐linked ubiquitination and stabilise GBP5. While Cys12 is a predicted palmitoylation site, we acknowledge that the C12S mutation may also affect other cysteine‐dependent post‐transcriptional modifications, and thus we refer to this residue as being required for ZDHHC14‐dependent regulation of GBP5 rather than definitively assigning it as the sole palmitoylation site.

**FIGURE 5:**
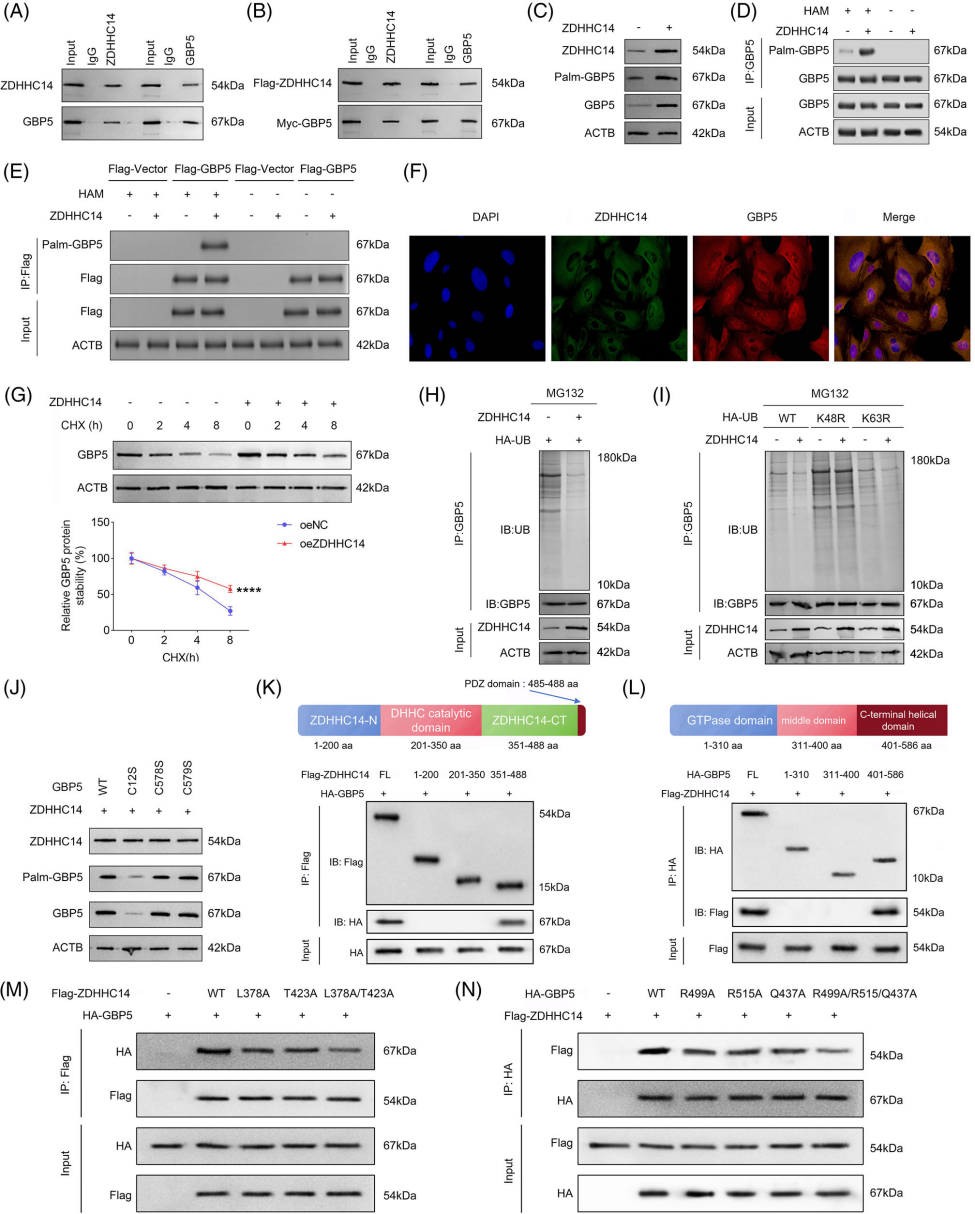
ZDHHC14 enhances GBP5 protein stability through Cys12‐dependent modification and by suppressing K48‐linked ubiquitination degradation. (A and B) Co‐IP experiments showed the interaction between Zdhhc14 and GBP5 in HEK293T cells. (C) Palmitoylation level and protein levels of GBP5 in HEK293T cells with Zdhhc14 overexpression detected by Western blot. (D and E) The palmitoylation of endogenous GBP5 and Flag‐tagged GBP5 was analysed by ABE assay. (F) IF staining showed co‐localisation of Zdhhc14 and GBP5 in HEK293T cells. (G) GBP5 protein stability was detected by Western blot following CHX treatment at 0, 2, 4 and 8 h. (H) Ubiquitination level of GBP5 with Zdhhc14 overexpression detected by IP–Western blot. (I) Ubiquitination level of GBP5 with K48R and K63R mutants. (J) Palmitoylation level and protein expression of GBP5 with C12S, C578S and C579S mutants. (K) Schematic representation of Zdhhc14 domains and mapping of the GBP5‐binding domain on Zdhhc14. (L) Schematic representation of GBP5 domains and mapping of the Zdhhc14‐binding domain on GBP5. (M) Co‐IP validation of Zdhhc14 point mutants (L378A, T423A) on GBP5 binding. (N) Co‐IP validation of GBP5 point mutants (R499A, R515A, Q437A) on Zdhhc14 binding. ****p < .0001.

To evaluate the structural plausibility of Cys12 as a palmitoylation site, we performed in silico structural analyses of GBP5 (Figure S6E–K and Table S2). A high‐confidence AlphaFold3 model of mouse GBP5 was obtained (mean pLDDT = 73, pTM = .564), and the PAE matrix together with MSA coverage supported a reliable overall topology, with the N‐terminal segment showing the expected flexibility (Figure S6E). Mapping of the three candidate cysteines onto the structure showed that Cys12 resides in the N‐terminal flexible loop, whereas Cys578 and Cys579 are spatially adjacent to each other in a distal region (Figure S6F). Cys12 displayed moderate solvent accessibility (relative SASA = .52), in contrast to the highly exposed Cys578 and Cys579 (both.95) (Figure S6G), and was located in a coil/loop element lacking rigid secondary‐structure constraints, adjacent to a predicted disordered loop (Figure S6H). Notably, Cys12 is embedded within the predicted membrane‐interacting region of GBP5, whereas Cys578 and Cys579 are located in a non‐membrane‐associated region (Figure S6I). ConSurf analysis revealed that Cys12 is highly evolutionarily conserved (grade 8–9), whereas Cys578 and Cys579 are highly variable (grade 1–4) (Figure S6J). Computational prediction of cysteine modifications further indicated that Cys12 carries a substantial S‐palmitoylation probability (31.35%) and high oxidation‐related scores, but negligible S‐nitrosylation and S‐glutathionylation probabilities (Table S2). Finally, comparison of unmodified and C12‐palmitoylated GBP5 models revealed that the palmitoyl chain at Cys12 folds back toward the N‐terminal loop and establishes hydrophobic contacts involving Leu13, Ile14, Gly15 and Asn24 (Figure S6K). Together, these structural analyses support Cys12 as a relatively favourable, evolutionarily conserved and likely regulated palmitoylation site, and suggest that its influence on GBP5 ubiquitination is more plausibly mediated through altered membrane localisation or local micro‐environment than through a defined conformational rearrangement; the palmitoylation–ubiquitination crosstalk is therefore presented as a proposed model.

To map the interacting domains, ZDHHC14 was divided into three regions: N‐terminal domain (1–200 aa), DHHC catalytic domain (201–350 aa) and C‐terminal domain (351–488 aa). Truncated mutants of each domain were constructed and subjected to IP assays. The results showed that HA–GBP5 co‐precipitated with full‐length Flag–ZDHHC14 and its C‐terminal fragment spanning residues 351–488, but not with the N‐terminal (1–200) or DHHC catalytic (201–350) domains. These results map the GBP5‐binding region to aa 351–488 of ZDHHC14 (Figure 5K). Similarly, GBP5 was divided into three domains: GTPase domain (1–310 aa), middle domain (311–400 aa) and C‐terminal helical domain (401–586 aa). Truncated mutants of each domain were constructed and subjected to IP assays. The results demonstrated that the C‐terminal helical domain of GBP5 was responsible for binding to ZDHHC14 (Figure 5L). Molecular dynamics simulations were further performed to validate the binding interface at the residue level (Figure S6). The simulations revealed that ZDHHC14 possesses two key binding sites and GBP5 possesses three key binding sites for their interaction. To further verify these binding sites, point mutants of ZDHHC14 (L378A, T423A) and GBP5 (R499A, R515A, Q437A) were generated and subjected to Co‐IP assays. The results showed that mutation of these residues significantly impaired the interaction between ZDHHC14 and GBP5 (Figure 5M,N). To determine whether ZDHHC14 promotes GBP5 palmitoylation through its intrinsic palmitoyltransferase activity, we generated a catalytically inactive ZDHHC14 mutant (DHHC‐dead) and compared it with wild‐type ZDHHC14. As shown in Figure S7, the DHHC‐dead mutant failed to suppress K48‐linked ubiquitination of GBP5, which instead accumulated to higher levels (Figure S7A); it also markedly reduced GBP5 palmitoylation (Figure S7B) and accelerated GBP5 degradation (Figure S7C). These results indicate that the DHHC catalytic activity of ZDHHC14 is required for GBP5 palmitoylation and stabilisation. Collectively, these findings demonstrate that ZDHHC14 facilitates GBP5 palmitoylation and inhibits its K48‐linked ubiquitination, thereby enhancing GBP5 protein stability.

### 3.6 GBP5 knockdown suppresses L‐OHP‐induced OIPN phenotypes and pyroptosis

Subsequently, we evaluated the functional role of GBP5 in L‐OHP‐treated mice. We administered AAV vectors carrying shGBP5 or a GBP5 overexpression plasmid to mice to achieve GBP5 knockdown or overexpression, respectively. Both treatments successfully modulated GBP5 protein levels in DRG tissues, as confirmed by Western blot (Figure 6A). To verify the cellular specificity of AAV9‐mediated transduction in the DRG, we performed immunofluorescence staining for GBP5 and the neuronal marker NeuN. As shown in Figure S8, GBP5 fluorescence signal was predominantly co‐localised with NeuN‐positive neurons in DRG sections from AAV9–shGBP5‐injected mice, demonstrating efficient neuronal targeting of the AAV9 vectors following intramuscular administration. Compared with the WT + AAV–shNC group, GBP5 knockdown significantly increased the PWT, indicating reduced mechanical hyperalgesia. Notably, the PWT in the Zdhhc14^−/−^ + AAV–GBP5 group was decreased compared with the Zdhhc14^−/−^ + AAV–NC group, demonstrating that GBP5 overexpression reverses the protective effects of Zdhhc14 deletion (Figure 6B). Consistently, cold sensitivity assessment showed that GBP5 knockdown markedly attenuated L‐OHP‐induced cold allodynia, with the WT + AAV–shGBP5 group exhibiting significantly reduced nocifensive duration compared with the WT + AAV–shNC group, whereas GBP5 overexpression in Zdhhc14^−/−^ mice prolonged the nocifensive duration relative to the Zdhhc14^−/−^ + AAV–NC group (Figure 6C). HE staining revealed severe DRG tissue damage in the WT + AAV–shNC group. In contrast, the WT + AAV–shGBP5 group exhibited significantly attenuated DRG injury, while the Zdhhc14^−/−^ + AAV–GBP5 group showed exacerbated damage, consistent with the PWT trends (Figure 6D). These findings suggest that GBP5 knockdown alleviates L‐OHP‐induced cold allodynia, mechanical hyperalgesia and DRG damage, whereas GBP5 overexpression counteracts the protective effects of Zdhhc14 deletion. ELISA revealed reduced plasma and DRG concentrations of IL‐18, IL‐1β, IL‐6 and TNF‐α in the WT + AAV–shGBP5 group compared with the WT + AAV–shNC group. Conversely, these levels were increased in the Zdhhc14^−/−^ + AAV–GBP5 group (Figure 6E–L). Furthermore, NLRP3, GSDMD‐N and CL‐CASP1 protein levels were down‐regulated in the WT + AAV–shGBP5 group. In contrast, these proteins were up‐regulated in the Zdhhc14^−/−^ + AAV–GBP5 group relative to the Zdhhc14^−/−^ + AAV–NC group (Figure 6M). Collectively, these results indicate that GBP5 knockdown significantly mitigates L‐OHP‐induced mechanical hyperalgesia, neuropathic pain, inflammatory activation and pyroptosis in male mice, suggesting a potential target for mechanistic intervention. Moreover, GBP5 overexpression in Zdhhc14‐deficient mice reverses these protective effects, confirming that GBP5 serves as a critical downstream effector of Zdhhc14 in regulating OIPN pathogenesis.

**FIGURE 6:**
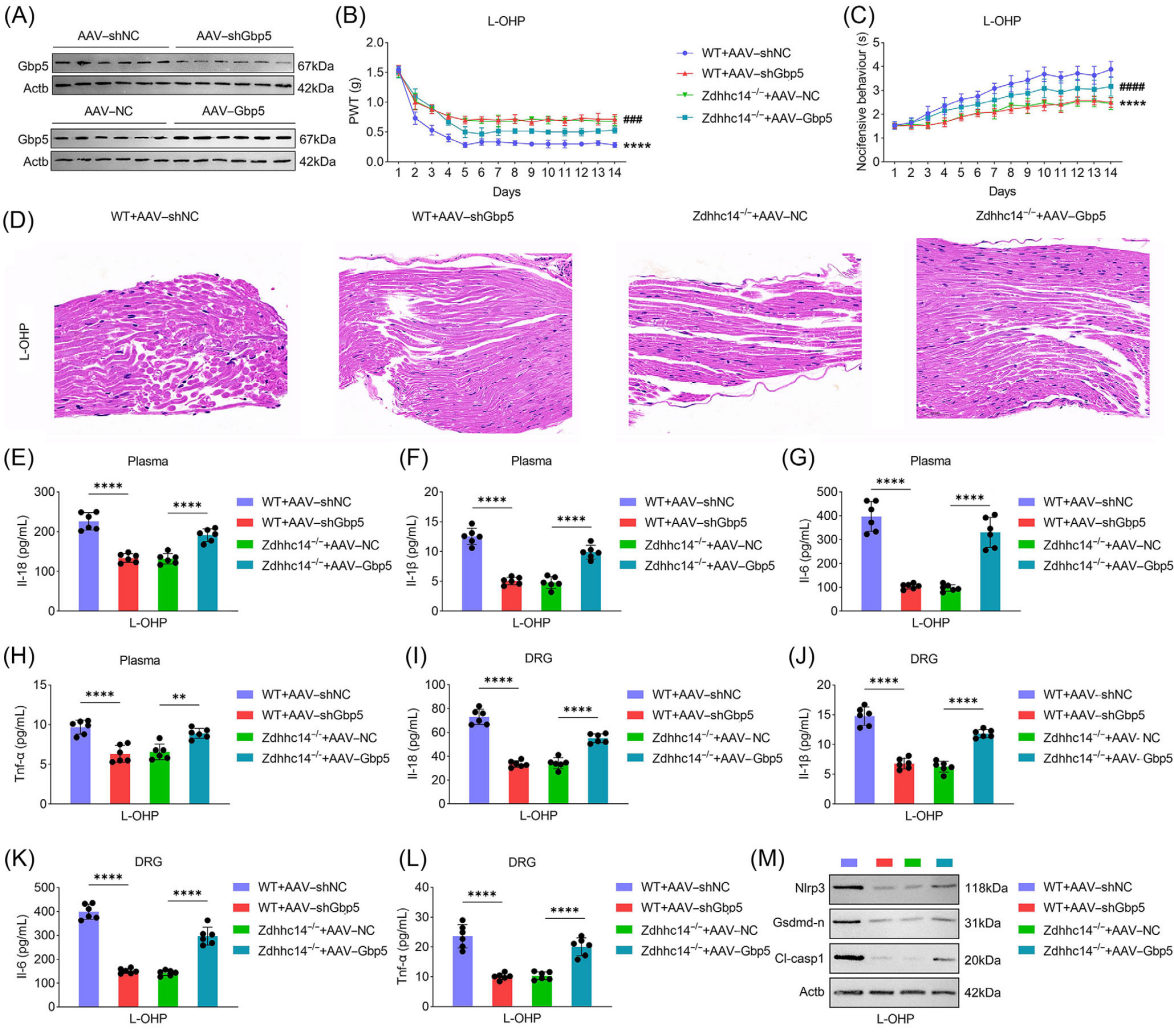
GBP5 knockdown suppresses L‐OHP‐induced OIPN phenotypes and pyroptosis. (A) The protein level of GBP5 in DRG tissues of mice detected by Western blot. (B) PWT of mice over 14 days. Two‐way repeated‐measures ANOVA with Bonferroni post‐hoc test; *n* = 6 mice per group (biological replicates). (C) Evaluation of cold allodynia in mice over the 14‐day observation period. Two‐way repeated‐measures ANOVA with Bonferroni post‐hoc test; *n* = 6 mice per group (biological replicates). Data are means ± SD. (D) HE staining of DRG tissues was performed to detect histopathological changes. (E–H) IL‐18, IL‐1β, IL‐6 and TNF‐α levels in plasma detected by ELISA. (I–L) IL‐18, IL‐1β, IL‐6 and TNF‐α levels in DRG tissues detected by ELISA. (M) The protein expression levels of NLRP3, GSDMD‐N and CL‐CASP1 in DRG tissues detected by Western blot. **p < .01 and ****p < .0001.

### 3.7 GBP5 overexpression reverses the inhibitory effects of Zdhhc14 knockdown on L‐OHP‐induced pyroptosis in DRG neuron‐enriched cultures

The role of GBP5 was further validated in L‐OHP‐treated DRG neuron‐enriched cultures. Western blot analysis confirmed that GBP5 overexpression significantly elevated GBP5 protein levels in cultured neurons (Figure 7A). Relative to the shZdhhc14 + oeNC control, the shZdhhc14 + oeGBP5 group displayed greater LDH efflux, higher IL‑18/IL‑1β, enhanced pyroptosis and up‐regulated NLRP3, GSDMD‑N and CL‑CASP1 (Figure 7B–G). Furthermore, the enhanced LDH release, inflammatory responses and pyroptosis induced by Zdhhc14 overexpression were suppressed by GBP5 knockdown (Figure S9A–G). These results collectively demonstrate that GBP5 serves as a critical downstream effector of Zdhhc14 in mediating L‐OHP‐induced pyroptosis and neuroinflammation in DRG neurons, and that reciprocal modulation of GBP5 expression bidirectionally regulates the functional outcomes driven by Zdhhc14.

**FIGURE 7:**
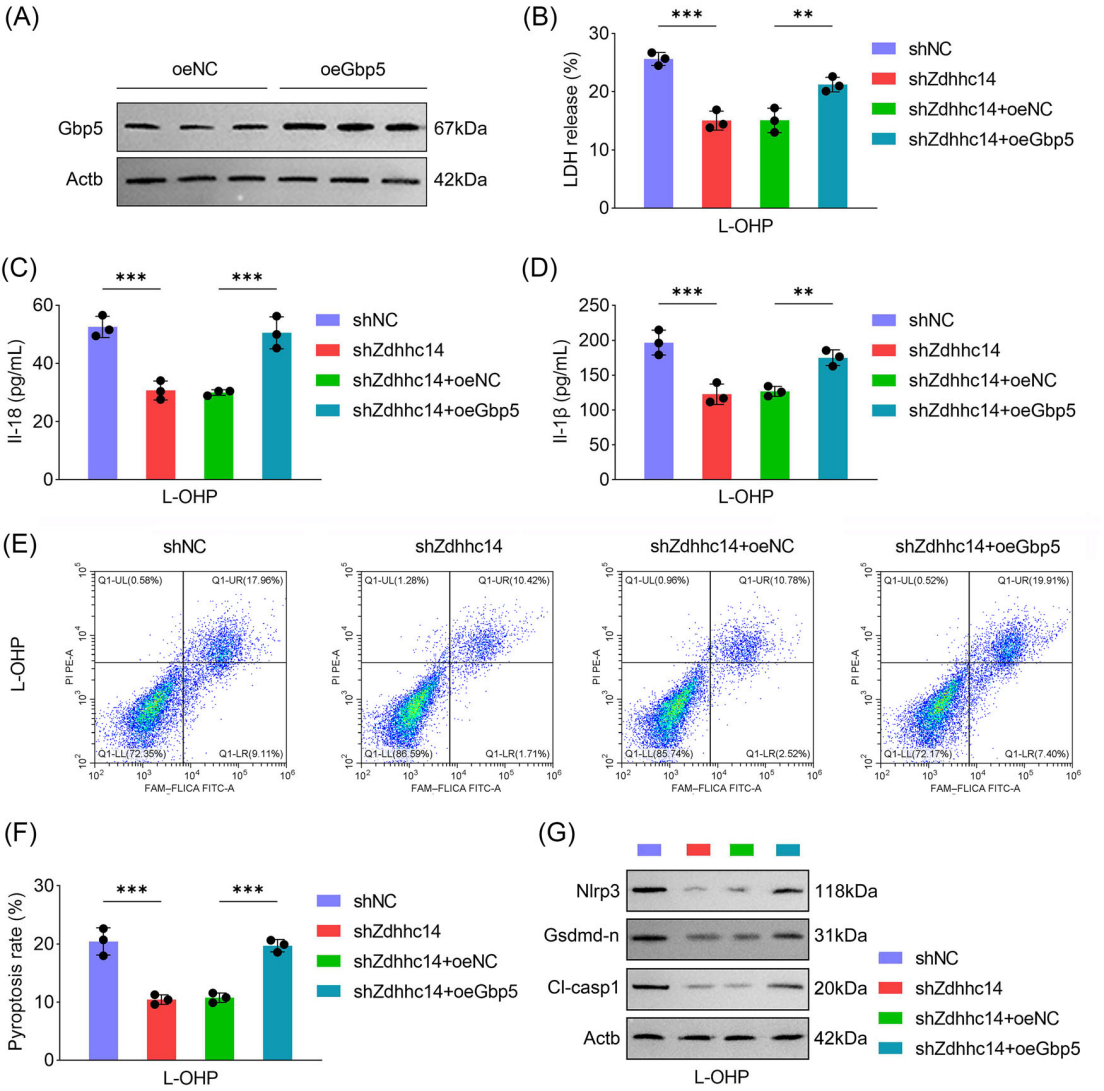
GBP5 overexpression reverses the inhibitory effects of Zdhhc14 knockdown on L‐OHP‐induced pyroptosis in DRG neuron‐enriched cultures. (A) The protein levels of GBP5 in DRG neuron‐enriched cultures after overexpression detected by Western blot. (B) LDH release rate of DRG neuron‐enriched cultures. (C and D) IL‐18 and IL‐1β levels in DRG neuron‐enriched cultures detected by ELISA. (E) Representative flow cytometry plots and (F) quantitative analysis of pyroptosis rate. (G) Protein expression levels of NLRP3, GSDMD‐N and CL‐CASP1 in DRG neuron‐enriched cultures detected by Western blot. *p < .01, ***p < .001 and ****p < .0001.

### 3.8 Zdhhc14 promotes L‐OHP‐induced pyroptosis in DRG neurons via GBP5 palmitoylation

Subsequently, DRG neuron‐enriched cultures were transfected with Zdhhc14 overexpressing plasmids, leading to a significant up‐regulation of intracellular Zdhhc14 protein levels (Figure 8A). Relative to the oeNC‑transfected controls, the GBP5–WT + oeNC group displayed marked rises in LDH efflux, the inflammatory cytokines IL‑18 and IL‑1β, pyroptotic cell death and the protein abundance of NLRP3, GSDMD‑N and CL‑CASP1. Notably, Zdhhc14 overexpression in the GBP5–WT background further exacerbated these effects. In contrast, in the GBP5–MUT (palmitoylation‐deficient) background, Zdhhc14 overexpression failed to induce significant changes in these parameters compared with the GBP5–MUT + oeNC group (Figure 8B–G). These findings indicate that Zdhhc14 exacerbates L‐OHP‐induced DRG injury, inflammatory cytokine release and pyroptosis activation by catalysing the palmitoylation of GBP5. Importantly, mutation of the GBP5 palmitoylation sites markedly attenuated the pro‐pyroptotic effects of Zdhhc14, indicating that GBP5 palmitoylation is a key molecular mechanism underlying Zdhhc14‐mediated regulation of pyroptosis in the DRG in OIPN.

**FIGURE 8:**
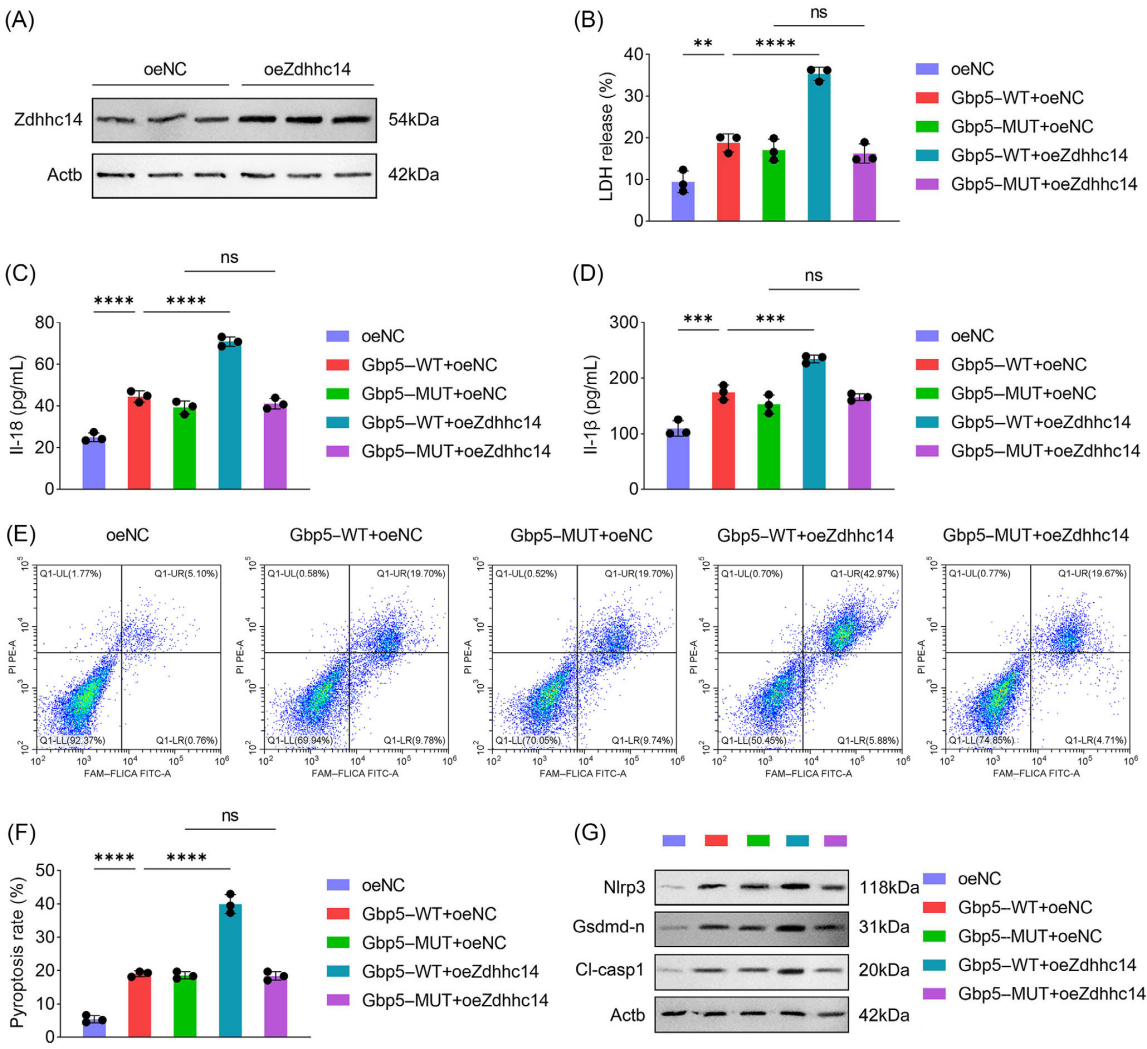
Zdhhc14 promotes L‐OHP‐induced pyroptosis in DRG neurons via GBP5 palmitoylation. (A) The protein levels of Zdhhc14 in DRG neuron‐enriched cultures after overexpression detected by Western blot. (B) LDH release rate of DRG neuron‐enriched cultures. (C and D) IL‐18 and IL‐1β levels in DRG neuron‐enriched cultures detected by ELISA. (E) Representative flow cytometry plots and (F) quantitative analysis of pyroptosis rate. (G) Protein expression levels of NLRP3, GSDMD‐N and CL‐CASP1 in DRG neuron‐enriched cultures detected by Western blot. ns, no significance, *p < .01, ***p < .001 and ****p < .0001.

## 4 DISCUSSION

Our present study demonstrated that Zdhhc14 expression increased in a time‐dependent manner in L‐OHP‐induced OIPN mouse models and was predominantly localised in DRG neurons. Zdhhc14 is a palmitoyl acyltransferase, and currently only limited studies have reported its role in the nervous system. In rat hippocampal neurons, Zdhhc14 deficiency leads to a significant increase in neuronal excitability.^26^ Zdhhc14 has also been shown to be up‐regulated in epilepsy models and exerts anticonvulsant protective effects by enhancing transient outward potassium currents to reduce neuronal excitability.^27^ Furthermore, Zdhhc14 has been demonstrated to be significantly associated with Parkinson's disease.^28^ Although previous studies have preliminarily revealed the involvement of Zdhhc14 in central nervous system diseases, our study is the first to link Zdhhc14 with OIPN, addressing a gap in the peripheral neuropathy literature. Moreover, we found that Zdhhc14 is primarily localised in DRG neurons, which echoes previous reports of Zdhhc14 expression patterns in hippocampal neurons, collectively suggesting the widespread distribution of Zdhhc14 and its potential regulatory role in neuronal function.

Furthermore, we observed that pyroptosis was significantly elevated in both L‐OHP‐induced OIPN mouse models and DRG neuron‐enriched cultures, accompanied by increased levels of systemic and neuroinflammation. Conversely, Zdhhc14 deficiency or knockdown significantly suppressed pyroptosis and inflammatory responses. These findings confirm that elevated pyroptosis in DRG neurons may be a critical driver of OIPN exacerbation and reveal that Zdhhc14 regulates pyroptosis in DRG neurons within the context of OIPN. Although VX‑765 is a widely used caspase‑1 inhibitor in pyroptosis research, we acknowledge that its specificity is not absolute. However, the consistent detection of GSDMD‑N – the terminal executor of pyroptosis – across all experimental groups provides the most direct evidence for GSDMD‑mediated pyroptosis in L‑OHP‑induced DRG neuronal injury. GSDMD‑N is recognised as the terminal effector and unique pyroptosis marker, differentiating this death from apoptosis and necroptosis.^29^, ^30^, ^31^ The role of pyroptosis in peripheral neuropathy has been previously investigated, particularly in diabetic peripheral neuropathy, where pyroptosis is markedly elevated in spinal dorsal horn microglia and Schwann cells, serving as a key factor driving neuropathy.^32^, ^33^ Moreover, a comparable pyroptosis elevation is seen in rodent neuropathic pain models after peripheral nerve lesion.^34^, ^35^ Notably, Chen et al.^15^ and Yang et al.^14^ used L‐OHP‐induced OIPN animal models and identified increased pyroptosis in microglia and DRG macrophages, respectively. These results further confirm the driving role of increased pyroptosis in OIPN but remain primarily focused on non‐neuronal cell types. In contrast, our study provides evidence supporting a role for pyroptosis within DRG neurons in OIPN, while acknowledging that this neuronal contribution is observed in the context of global Zdhhc14 deletion rather than sensory‐neuron‐specific manipulation. This finding extends the research perspective from the neuroimmune microenvironment to potentially intrinsic neuronal injury mechanisms, providing a more comprehensive cytological basis for understanding the pathophysiological processes of OIPN.

Furthermore, we demonstrated that Zdhhc14 enhances GBP5 protein stability and suppresses its ubiquitination‐mediated degradation through a Cys12‐dependent mechanism. While bioinformatic prediction and mutational analysis implicate Cys12 as a palmitoylation site, we acknowledge that cysteine residues can undergo multiple post‐translational modifications including oxidation, S‐nitrosylation, S‐glutathionylation and persulfidation, and therefore the C12S mutation may affect modifications beyond palmitoylation. Palmitoylation has been established as a pivotal regulatory mechanism in peripheral neuropathy. In diabetic peripheral neuropathy, enhanced palmitoylation of substrate proteins TSHR and PEX11B within Schwann cells has been shown to drive neuropathy progression.^21^, ^36^ Additionally, Cao et al.^22^ identified PRDX6 is a critical linker between S‐palmitoylation and diabetic neuropathy, further underscoring the broad pathogenic involvement of dysregulated palmitoylation machinery in peripheral nerve disorders. Notably, Zhang et al.^37^ similarly observed increased palmitoylation in DRG neurons in an L‐OHP‐induced OIPN rat model, where inhibition of palmitoylation or knockdown of its substrate δ‐catenin alleviated pain. Consistent with this study, we confirmed the driving role of palmitoylation in OIPN progression, providing the first evidence that Zdhhc14‐mediated palmitoylation of GBP5 at C12 contributes to OIPN progression by facilitating DRG neuronal pyroptosis. This not only unveils a novel mechanism by which palmitoylation drives OIPN but also identifies a new regulatory site for Zdhhc14‐mediated GBP5 palmitoylation.

Recent studies have demonstrated that GBP5, as a key activator of the NLRP3 inflammasome, plays a central role in inflammatory responses and pyroptosis across multiple disease models.^38^, ^39^, ^40^ Its pro‐inflammatory function has been extensively validated in both immune cells and parenchymal cells.^41^, ^42^ However, GBP5 activity is subject to complex regulation, including transcriptional suppression and protein‐level interactions.^41^ Our study reveals that in the OIPN model, the pro‐pyroptotic function of GBP5 is regulated by palmitoylation. Zdhhc14 enhances GBP5 protein stability by promoting its palmitoylation, thereby amplifying NLRP3 inflammasome‐mediated neuronal pyroptosis. Furthermore, GBP5 stability is also regulated by ubiquitination, and Zdhhc14 overexpression suppresses GBP5 ubiquitination‐mediated degradation. This study provides the first evidence of the regulatory role of palmitoylation–ubiquitination crosstalk in GBP5‐mediated neuronal pyroptosis, which expands the post‐translational modification regulatory network of GBP5 in neuroinflammatory diseases but also offers a new theoretical foundation for targeting the Zdhhc14/GBP5 axis in OIPN treatment. Beyond neurological disorders, S‐palmitoylation has also been implicated in the regulation of cancer stem cell properties in lung adenocarcinoma, underscoring the broad pathophysiological relevance of this post‐translational modification across different disease contexts.^43^

Nevertheless, some shortcomings of the present work ought to be recognised. Although we confirmed that Zdhhc14 mediates GBP5 palmitoylation at C12, the precise upstream signals that regulate Zdhhc14 expression in response to oxaliplatin stimulation remain to be fully elucidated. Moreover, while our findings establish the key role of the Zdhhc14/GBP5 axis in the DRG, the potential involvement of such cascades across distinct cellular contexts within the DRG, such as satellite glial cells, warrants further investigation. We acknowledge that GFAP and IBA1, used herein as markers for non‐neuronal DRG cells, have known limitations: GFAP can be up‐regulated in reactive glial states beyond satellite glial cells, and IBA1‐positive cells in the DRG may include resident macrophages or infiltrating monocyte‐derived cells rather than microglia. Future studies using additional cell‐type‐specific markers will be needed to refine these observations. Another limitation is that global Zdhhc14^−/−^ mice cannot exclude indirect effects from non‐neuronal cells, leaving the DRG neuron‐autonomous role unproven. Although prior studies indicate that Zdhhc14 knockout primarily affects non‐neural tissues, conditional knockout models are required to definitively confirm its cell‐autonomous function. We are currently planning DRG neuron‐specific conditional knockout experiments to address this in future work. Additionally, despite the robust cellular evidence supporting C12 as the functionally essential palmitoylation site of GBP5, the absence of in vivo site‐specific rescue experiments using C12S mutant mice represents a limitation of the present study. Future investigations employing AAV‐mediated delivery of GBP5–C12S into Zdhhc14 knockout animals will be required to definitively validate the indispensable role of C12 in GBP5 palmitoylation‐dependent pyroptosis regulation in vivo. Future studies using specific inhibitors or knockdown approaches targeting these candidate enzymes will be needed to definitively establish the specificity of Zdhhc14. Additionally, although our data identify the Zdhhc14/GBP5 axis as a mechanistic contributor to OIPN, and potentially druggable in principle, we did not evaluate whether targeting this pathway affects the anti‐tumour efficacy of oxaliplatin. Given that palmitoylation, GBP‐family proteins, and inflammasome/inflammatory cell‐death signalling may also influence tumour‐cell biology and the tumour immune microenvironment, it remains unknown whether Zdhhc14 or GBP5 suppression compromises oxaliplatin‐mediated tumour control. Future studies using tumour‐bearing mouse models comparing vehicle, oxaliplatin, target inhibition alone and combined treatment – with parallel assessment of tumour volume, tumour weight, body weight and neuropathic pain behaviours – will be required to establish the translational applicability of this pathway, and our findings should therefore be interpreted as preclinical mechanistic evidence rather than as a validated therapeutic strategy. Additionally, all animal experiments and primary DRG neuron isolations were performed exclusively in male mice to minimise potential confounding effects of hormonal fluctuations on nociceptive processing and neuroimmune interactions, and no key findings were validated in female animals. Therefore, our conclusions are restricted to male OIPN and may not be generalisable to females, in whom sex‐specific differences in inflammatory signalling and chemotherapy‐induced peripheral neuropathy phenotypes have been reported. Future studies should confirm the Zdhhc14/GBP5 mechanism in female mice to ensure broader translational relevance.

Collectively, we demonstrate that Zdhhc14 is up‐regulated in DRG of L‐OHP‐induced OIPN mice, where it promotes GBP5 palmitoylation and inhibits its ubiquitination‐mediated degradation, thereby enhancing GBP5 protein stability and consequently exacerbating DRG pyroptosis. In light of the limitations discussed above – including the absence of pharmacologically tractable Zdhhc14‐selective interventions, lack of validation in human sensory neurons or clinical OIPN material, exclusive use of male mice, non‐cell‐type‐specific genetic approaches and unknown effects on oxaliplatin anti‐tumour efficacy – we conclude that the Zdhhc14/GBP5 pathway represents a potential mechanistic target warranting further translational evaluation, rather than an immediately therapeutically tractable strategy.

## AUTHOR CONTRIBUTIONS

All authors participated in the design, interpretation of the studies and analysis of the data and review of the manuscript. X. J. and W. Q. L. drafted the work and revised it critically for important intellectual content; Y. Z. L. and W. S. were responsible for the acquisition, analysis and interpretation of data for the work; X. D. Z. and L. T. made substantial contributions to the guidance and supervision of the work. All authors read and approved the final manuscript.

## CONFLICT OF INTEREST STATEMENT

The authors declare no conflicts of interest.

## ETHICS STATEMENT

The animal study was approved by the Laboratory Animal Welfare and Ethics Committee of Renmin Hospital of Wuhan University. All animal experiments complied with the ARRIVE guidelines. All methods were carried out in accordance with relevant guidelines and regulations.

## Supporting information



Supporting information: ctm270874‐supp‐0001‐SuppMat.docx

Supporting information: ctm270874‐supp‐0002‐SuppMat.xlsx

Supporting information: ctm270874‐supp‐0003‐SuppMat.docx

## Data Availability

The datasets used and/or analysed during the current study are available from the corresponding author on reasonable request.

## References

[1] Tian F , Dai H , Sha D , et al. Total neoadjuvant treatment with short‐course radiotherapy followed by sintilimab plus capecitabine‐oxaliplatin versus short‐course radiotherapy followed by capecitabine‐oxaliplatin in patients with locally advanced rectal cancer (SPRING‐01): a single‐centre, open‐label, phase 2, randomised controlled trial. The Lancet Oncology. 2025;26:1043‐1054. doi:10.1016/S1470-2045(25)00286-4 40645192

[2] Wang X , Lu C , Wei B , et al. Perioperative versus adjuvant S‐1 plus oxaliplatin chemotherapy for stage II/III resectable gastric cancer (RESONANCE): a randomized, open‐label, phase 3 trial. J Hematol Oncol. 2024;17:17. doi:10.1186/s13045-024-01536-7 PMC11003079 38589926

[3] Srivastava J , EK DO , Sivakumar S , Mullaguri N . A case of folinic acid, fluorouracil and oxaliplatin (FOLFOX) chemotherapy regimen associated neurotoxicity and review of literature. BMC Neurol. 2025;25:481. doi:10.1186/s12883-025-04416-w PMC12648844 41291490

[4] Yang Y , Zhao B , Gao X , et al. Targeting strategies for oxaliplatin‐induced peripheral neuropathy: clinical syndrome, molecular basis, and drug development. J Exp Clin Cancer Res. 2021;40:331. doi:10.1186/s13046-021-02141-z PMC8532307 34686205

[5] Burgess J , Ferdousi M , Gosal D , et al. Chemotherapy‐induced peripheral neuropathy: epidemiology, pathomechanisms and treatment. Oncol Ther. 2021;9:385‐450. doi:10.1007/s40487-021-00168-y PMC8593126 34655433

[6] Teng C , Cohen J , Egger S , Blinman PL , Vardy JL . Systematic review of long‐term chemotherapy‐induced peripheral neuropathy (CIPN) following adjuvant oxaliplatin for colorectal cancer. Support Care Cancer. 2022;30:33‐47. doi:10.1007/s00520-021-06502-4 34410459

[7] Kang L , Tian Y , Xu S , Chen H . Oxaliplatin‐induced peripheral neuropathy: clinical features, mechanisms, prevention and treatment. J Neurol. 2021;268:3269‐3282. doi:10.1007/s00415-020-09942-w 32474658

[8] Xu J , Lu L , Jiang S , et al. Paeoniflorin ameliorates oxaliplatin‐induced peripheral neuropathy via inhibiting neuroinflammation through influence on gut microbiota. Eur J Pharmacol. 2024;971:176516. doi:10.1016/j.ejphar.2024.176516 38513881

[9] Illias AM , Yu K , Hwang S , et al. Dorsal root ganglion toll‐like receptor 4 signaling contributes to oxaliplatin‐induced peripheral neuropathy. Pain. 2022;163:923‐935. doi:10.1097/j.pain.0000000000002454 34490849

[10] Chen C , Xu J , Gu Z , et al. Danggui Sini decoction alleviates oxaliplatin‐induced peripheral neuropathy by regulating gut microbiota and potentially relieving neuroinflammation related metabolic disorder. Chin Med. 2024;19:58. doi:10.1186/s13020-024-00929-7 PMC10999090 38584284

[11] Shatunova S , Aktar R , Peiris M , Lee JYP , Vetter I , Starobova H . The role of the gut microbiome in neuroinflammation and chemotherapy‐induced peripheral neuropathy. Eur J Pharmacol. 2024;979:176818. doi:10.1016/j.ejphar.2024.176818 39029779

[12] Liu Y , Pan R , Ouyang Y , et al. Pyroptosis in health and disease: mechanisms, regulation and clinical perspective. Signal Transduct Target Ther. 2024;9:245. doi:10.1038/s41392-024-01958-2 PMC11413206 39300122

[13] Rao Z , Zhu Y , Yang P , et al. Pyroptosis in inflammatory diseases and cancer. Theranostics. 2022;12:4310‐4329. doi:10.7150/thno.71086 PMC9169370 35673561

[14] Yang Y , Zhao B , Huo J , et al. Microglial macrophage‐derived ds‐HMGB1 in DRG orchestrates neuropathic pain through immune‐neural signaling. Cell Rep. 2025;44:116671. doi:10.1016/j.celrep.2025.116671 41385369

[15] Chen R , Hu J , Zhang Y , et al. Total glucosides of paeony ameliorates chemotherapy‐induced neuropathic pain by suppressing microglia pyroptosis through the inhibition of KAT2A‐mediated p38 pathway activation and succinylation. Sci Rep. 2024;14:31875. doi:10.1038/s41598-024-83207-8 PMC11686281 39738348

[16] Wang Y , Ma H , Zhang B , et al. Protein palmitoylation in hepatic diseases: functional insights and therapeutic strategies. J Adv Res. 2025;76:307‐326. doi:10.1016/j.jare.2024.12.041 PMC12793770 39732335

[17] Chamberlain LH , Shipston MJ . The physiology of protein S‐acylation. Physiol Rev. 2015;95:341‐376. doi:10.1152/physrev.00032.2014 PMC4551212 25834228

[18] Peng J , Cai Z , Ouyang P , et al. GluA2 palmitoylation by SELENOK modulates AMPAR assembly and synaptic plasticity in Alzheimer's disease. Redox Biol. 2025;86:103831. doi:10.1016/j.redox.2025.103831 PMC12446660 40848510

[19] Virlogeux A , Scaramuzzino C , Lenoir S , et al. Increasing brain palmitoylation rescues behavior and neuropathology in Huntington disease mice. Sci Adv. 2021;7:b799. doi:10.1126/sciadv.abb0799 PMC8011966 33789888

[20] Fan X , Gong M , Zhang S , et al. Blocking palmitoylation of apelin receptor alleviates morphine tolerance in neuropathic cancer pain. Int J Biol Sci. 2024;20:47‐60. doi:10.7150/ijbs.86888 PMC10750292 38164190

[21] Yang YM , Ma HB , Xiong Y , Wu Q , Gao XK . PEX11B palmitoylation couples peroxisomal dysfunction with Schwann cells fail in diabetic neuropathy. J Biomed Sci. 2025;32:20. doi:10.1186/s12929-024-01115-5 PMC11818136 39934809

[22] Cao Y , Wang W , Zhan X , Zhang Y . PRDX6: a protein bridging S‐palmitoylation and diabetic neuropathy. Front Endocrinol (Lausanne). 2022;13:992875. doi:10.3389/fendo.2022.992875 PMC9478578 36120430

[23] Greaves J , Chamberlain LH . New links between S‐acylation and cancer. J Pathol. 2014;233:4‐06. doi:10.1002/path.4339 24615251

[24] Lin T , Hu L , Hu F , et al. NET‐triggered NLRP3 activation and IL18 release drive oxaliplatin‐induced peripheral neuropathy. Cancer Immunol Res. 2022;10:1542‐1558. doi:10.1158/2326-6066.CIR-22-0197 PMC9716254 36255412

[25] Osonoi S , Mizukami H , Takeuchi Y , et al. RAGE activation in macrophages and development of experimental diabetic polyneuropathy. JCI insight. 2022;7:e160555. doi:10.1172/jci.insight.160555 PMC9746912 36477360

[26] Sanders SS , Hernandez LM , Soh H , et al. The palmitoyl acyltransferase ZDHHC14 controls Kv1‐family potassium channel clustering at the axon initial segment. Elife. 2020;9:e56058. doi:10.7554/eLife.56058 PMC7685708 33185190

[27] Shen Q , Liu N , Jiang Y , Liu L , Hou X . Decreased neuronal excitability in hypertriglyceridemia hamsters with acute seizures. Front Neurol. 2024;15:1500737. doi:10.3389/fneur.2024.1500737 PMC11730077 39811454

[28] Qiu J , Yang J , Zhu R , Xian D , Yang K . The causal relationship between palmitoylation genes and Parkinson's disease: a mendelian randomization study. J Mol Neurosci. 2025;75:143. doi:10.1007/s12031-025-02436-2 41120779

[29] Zhu C , Xu S , Jiang R , Yu Y , Bian J , Zou Z . The gasdermin family: emerging therapeutic targets in diseases. Signal Transduct Target Ther. 2024;9:87. doi:10.1038/s41392-024-01801-8 PMC10999458 38584157

[30] Rühl S , Broz P . Regulation of lytic and non‐lytic functions of gasdermin pores. J Mol Biol. 2022;434:167246. doi:10.1016/j.jmb.2021.167246 34537232

[31] Su K , Shen C , Wu W , Zhou F , Ye H . Post‐translational modifications of gasdermin D: implications for pyroptosis and autoimmune diseases. Int Immunopharmacol. 2026;179:116605. doi:10.1016/j.intimp.2026.116605 41946127

[32] Liao Q , Yang Y , Li Y , et al. Targeting TANK‐binding kinase 1 attenuates painful diabetic neuropathy via inhibiting microglia pyroptosis. Cell Commun Signal. 2024;22:368. doi:10.1186/s12964-024-01723-6 PMC11264750 39030571

[33] Feng H , Wu T , Chin J , et al. Tangzu granule alleviate neuroinflammation in diabetic peripheral neuropathy by suppressing pyroptosis through P2×7R /NLRP3 signaling pathway. J Ethnopharmacol. 2025;337:118792. doi:10.1016/j.jep.2024.118792 39251151

[34] Wang J , Chen P , Han G , et al. Rab32 facilitates Schwann cell pyroptosis in rats following peripheral nerve injury by elevating ROS levels. J Transl Med. 2024;22:194. doi:10.1186/s12967-024-04999-x PMC10885539 38388913

[35] Shin J , Wu J , Park H , et al. Microglial pyroptosis drives neuropathic pain and targeting NLRP3 alleviates pain and neuroinflammation. Biochim Biophys Acta Mol Basis Dis 2025;1871:167737. doi:10.1016/j.bbadis.2025.167737 39971256

[36] Fan J , Pan Q , Gao Q , Li W , Xiao F , Guo L . TSH combined with TSHR aggravates diabetic peripheral neuropathy by promoting oxidative stress and apoptosis in schwann cells. Oxid Med Cell Longev. 2021;2021:2482453. doi:10.1155/2021/2482453 PMC8601831 34804362

[37] Zhang X , Ding H , Xu T , et al. Palmitoylation of δ‐catenin promotes kinesin‐mediated membrane trafficking of Na(v)1.6 in sensory neurons to promote neuropathic pain. Sci Signal. 2018;11:r4394. doi:10.1126/scisignal.aar4394 29588412

[38] Elmadbouh I , Singla DK . BMP‐7 attenuates inflammation‐induced pyroptosis and improves cardiac repair in diabetic cardiomyopathy. Cells. 2021;10:2640. doi:10.3390/cells10102640 PMC8533936 34685620

[39] Chen Z , Su Y , Liu G , et al. RETN exacerbates sepsis by GBP5/NLRP3 signaling pathway‐mediated pyroptosis of macrophage. Genes Immun. 2026; 27:223‐235. doi:10.1038/s41435-026-00387-1 41764353

[40] Li Y , Lin X , Wang W , et al. The proinflammatory role of guanylate‐binding protein 5 in inflammatory bowel diseases. Front Microbiol. 2022;13:926915. doi:10.3389/fmicb.2022.926915 PMC9201962 35722277

[41] Qiu J , Che Q , Zhang Y , et al. Nuclear receptor ERRγ protects against cardiac ischemic injury by suppressing GBP5‐mediated myocardial inflammation. FASEB J. 2025;39:e70819. doi:10.1096/fj.202500763R PMC12242898 40636987

[42] Zou C , Shen J , Xu F , Ye Y , Wu Y , Xu S . Immunoreactive microenvironment modulator GBP5 suppresses ovarian cancer progression by inducing canonical pyroptosis. J Cancer. 2024;15:3510‐3530. doi:10.7150/jca.94616 PMC11134437 38817865

[43] Zhang Y , Li F , Fu K , Liu X , Lien I , Li H . Potential role of S‐palmitoylation in cancer stem cells of lung adenocarcinoma. Front Cell Dev Biol. 2021;9:734897. doi:10.3389/fcell.2021.734897 PMC8490697 34621750

