# Supplementary material for: Zdhhc14 promotes pyroptosis in the dorsal root ganglion of oxaliplatin‐induced peripheral neuropathy mice by enhancing GBP5 palmitoylation and suppressing its ubiquitination
Source: Clin Transl Med. 2026 Nov 4;16(11):e70874. doi: 10.1002/ctm2.70874 (PMC13733372; doi:10.1002/ctm2.70874)
Supplement: Supplementary file 1 [file CTM2-16-e70874-s002.docx]

Table S1. Identification of interacting proteins by IP-MS

| **Accession** | **Gene name** | **# AAs** | **MW [kDa]** | **calc. pI** | **Abundance** |
| --- | --- | --- | --- | --- | --- |
| **Q8CFB4** | **GBP5** | **590** | **66.9** | **7.39** | **899073.6875** |
| Q8C3X4 | GUF1 | 669 | 74.3 | 8.59 | 857462.8125 |
| Q9WUU9 | MCM3AP | 1980 | 218.3 | 6.39 | 518559.9375 |
| Q8C3R1 | BRAT1 | 821 | 88.1 | 5.27 | 485205.4375 |
| Q8VDW0 | DDX39A | 427 | 49.1 | 5.68 | 459345.0313 |
| Q5RL79 | KRTCAP2 | 114 | 12.5 | 9.61 | 457256.0625 |
| Q8VCL2 | SCO2 | 266 | 29.8 | 8.85 | 329923.6875 |
| Q921U8 | SMTN | 971 | 104.8 | 8.73 | 262087.39 |
| Q8BR65 | SUDS3 | 328 | 38.1 | 5.66 | 214492.875 |
| Q8N7N5 | DCAF8 | 417 | 46.5 | 5.26 | 169912.3594 |

Table S2. Structural and evolutionary annotation of cysteine residues in GBP5 with predicted modifications and putative functions

| Site | Spatial location and secondary structure | Relative SASA | Evolutionary conservation (ConSurf) | Predicted multi-modification features | Functional inference |
| --- | --- | --- | --- | --- | --- |
| Cys12 | N-terminal flexible loop region (loop), adjacent to a disordered loop; located in the membrane-interaction region | 0.52 | Highly conserved (8-9, purple-red) | S-palmitoylation 31.35%，S-nitrosylation 0.01%, S-sulfenylation 89.27%, S-sulfhydration 43.18%, S-sulfinylation 90.25%   S-glutathionylation 0.00% | Key regulatory site; may mediate membrane anchoring |
| Cys578 | Core domain surface, non-membrane-interacting region | 0.95 | Low conservation/variable (1-4, light blue/white) | S-glutathionylation 0.00% | Species-specific function or redundancy |
| Cys579 | Core domain surface, adjacent to Cys57 | 0.95 | Low conservation/variable (1-4, light blue/white) | S-glutathionylation 0.00% | Species-specific function or redundancy |

Figure S1 Optimization of L-OHP concentration and exposure duration for the establishment of an in vitro OIPN model

(A) Cell viability after treatment with different doses of L-OHP on primary DRG cells. (B) Cell viability of primary DRG cells after treatment with 0.1 μg/ml L-OHP for different durations.

Figure S2 Representative MS/MS spectrum of a GBP5-derived peptide identified by IP-MS

Figure S3 Western blot was performed to detect the effect of (A) ZDHHC1 and (B) ZDHHC16 on the protein and palmoylation levels in HEK293T cells.

Figure S4 Ubiquitination level of GBP5 with K48R and K63R mutants in mouse DRG tissues

Figure S5 The C12S mutation abolishes ZDHHC14-mediated suppression of K48-linked ubiquitination and protein stabilization of GBP5.

(A) HEK293T cells were co-transfected with HA-ubiquitin, ZDHHC14 (or empty vector), and either GBP5-WT or GBP5-C12S, followed by MG132 treatment. IP with anti-GBP5 and western blotting with anti-ubiquitin or anti-K48-ubiquitin (K48-UB) were performed. (B) Western blot analysis of GBP5 protein levels in cells transfected as in (A). (C) CHX chase assay showing GBP5 protein stability over 8 h in cells expressing GBP5-WT or GBP5-C12S, with or without ZDHHC14 co-expression. ****P<0.0001.

Figure S6 Structural analyses of GBP5 cysteine residues and molecular dynamics simulations of the Zdhhc14-GBP5 interaction interface.

(A) Root mean square deviation (RMSD) trajectories of Zdhhc14 (black), GBP5 (red), and the Zdhhc14-GBP5 complex (blue) over a 100 ns simulation. (B) Root mean square fluctuation (RMSF) profiles of Zdhhc14 and GBP5 residues. (C) The number of intermolecular hydrogen bonds formed between Zdhhc14 and GBP5 during the simulation. (D) Per-residue energy decomposition analysis identifying key residues contributing to the binding affinity. (E) Quality assessment of the AlphaFold3-predicted mouse GBP5 structure. Left: predicted aligned error (PAE) matrix, with darker green indicating lower expected position error; right: multiple-sequence-alignment (MSA) coverage and sequence identity to the query. Model confidence metrics (mean pLDDT = 73; pTM = 0.564) support a reliable overall fold, with the N-terminal region showing higher predicted error consistent with its intrinsic flexibility. (F) Spatial mapping of Cys12, Cys578 and Cys579 onto the three-dimensional structure of GBP5. Left: overall surface model; right: zoomed view with cysteine side chains shown as sticks (sulfur atoms in yellow). Cys12 is located in the N-terminal region, whereas Cys578 and Cys579 are spatially adjacent to each other in a distal region. (G) Solvent-accessible surface area (SASA) analysis of the three cysteine residues. Normalized relative SASA values are indicated in red: Cys12 = 0.52 (moderately exposed); Cys578 = 0.95 and Cys579 = 0.95 (highly exposed). (H) Secondary-structure context of Cys12. Loops (salmon), α-helices (cyan) and β-sheets (magenta) are colored as indicated. Cys12 is located in a coil/loop element at the N-terminus, adjacent to a predicted disordered loop (dashed circle), lacking rigid secondary-structure constraints. The upper right panel shows a zoomed view of the Cys12 region.

(I) Proximity of Cys12 to the predicted membrane-interacting region of GBP5. The membrane-interacting region is shown in yellow and Cys12 in red; Cys578 and Cys579 are located in a distal, non-membrane-associated region. Right panels: zoomed ribbon (top) and surface (bottom) views of the Cys12 microenvironment within the membrane-interacting region. (J) Evolutionary conservation analysis of GBP5 by ConSurf. Residues are colored by conservation grade (1, variable, teal; 9, conserved, maroon). Cys12 is located in a highly conserved region (grade 8–9, lower left zoom), whereas Cys578 and Cys579 are highly variable (grade 1–4, lower right zoom). (K) Local micro-environment of Cys12 in unmodified (left) and C12-palmitoylated (right) GBP5 models (AlphaFold3; CCD_P1L modification at Cys12). In the unmodified model, the Cys12 side chain points toward Asn24; in the C12-palmitoylated model, the palmitoyl chain folds back toward the N-terminal loop and establishes hydrophobic contacts involving Leu13, Ile14, Gly15 and Asn24.

Figure S7 Catalytic activity of Zdhhc14 is required for GBP5 palmitoylation and stabilization.

(A) HEK293T cells were co-transfected with Flag-tagged Zdhhc14 wild-type (WT) or catalytically inactive mutant (DHHC-dead), HA-ubiquitin, and GBP5, followed by MG132 treatment. Immunoprecipitation with anti-GBP5 and western blotting with anti-ubiquitin or anti-K48-ubiquitin (K48-UB) were performed. (B) ABE assay detecting GBP5 palmitoylation (Palm-GBP5) and protein levels in cells expressing Zdhhc14-WT or DHHC-dead mutant. (C) CHX chase assay showing GBP5 protein stability over 8 h in cells expressing Zdhhc14-WT or DHHC-dead mutant. ****P<0.0001.

Figure S8 Immunofluorescence co-localization of GBP5 with neuronal marker NeuN in dorsal root ganglion sections from AAV9-shGBP5-injected mice

Figure S9 GBP5 knockdown reverses the promotion of Zdhhc14 overexpression on L-OHP-induced pyroptosis in primary DRG neurons

(A) The protein levels of GBP5 in primary DRG neurons after overexpression detected by western blot. (B) LDH release rate of primary DRG neurons. (C-D) IL-18 and IL-1β levels in primary DRG neurons detected by ELISA. (E) Representative flow cytometry plots and (F) quantitative analysis of pyroptosis rate. (G) Protein expression levels of NLRP3, GSDMD-N, and CL-CASP1 in primary DRG neurons detected by Western blot. ***P<0.001 and ****P<0.0001.
