# Supplementary material for: Zdhhc14 promotes pyroptosis in the dorsal root ganglion of oxaliplatin‐induced peripheral neuropathy mice by enhancing GBP5 palmitoylation and suppressing its ubiquitination
Source: Clin Transl Med. 2026 Nov 4;16(11):e70874. doi: 10.1002/ctm2.70874 (PMC13733372; doi:10.1002/ctm2.70874)
Supplement: Supplementary file 2 [file CTM2-16-e70874-s003.docx]

Table S1. Identification of interacting proteins by IP-MS

| **Accession** | **Gene name** | **# AAs** | **MW [kDa]** | **calc. pI** | **Abundance** |
| --- | --- | --- | --- | --- | --- |
| **Q8CFB4** | **GBP5** | **590** | **66.9** | **7.39** | **899073.6875** |
| Q8C3X4 | GUF1 | 669 | 74.3 | 8.59 | 857462.8125 |
| Q9WUU9 | MCM3AP | 1980 | 218.3 | 6.39 | 518559.9375 |
| Q8C3R1 | BRAT1 | 821 | 88.1 | 5.27 | 485205.4375 |
| Q8VDW0 | DDX39A | 427 | 49.1 | 5.68 | 459345.0313 |
| Q5RL79 | KRTCAP2 | 114 | 12.5 | 9.61 | 457256.0625 |
| Q8VCL2 | SCO2 | 266 | 29.8 | 8.85 | 329923.6875 |
| Q921U8 | SMTN | 971 | 104.8 | 8.73 | 262087.39 |
| Q8BR65 | SUDS3 | 328 | 38.1 | 5.66 | 214492.875 |
| Q8N7N5 | DCAF8 | 417 | 46.5 | 5.26 | 169912.3594 |

Figure S1 Optimization of L-OHP concentration and exposure duration for the establishment of an in vitro OIPN model

(A) Cell viability after treatment with different doses of L-OHP on primary DRG cells. (B) Cell viability of primary DRG cells after treatment with 0.1 μg/ml L-OHP for different durations.

Figure S2 Abundance visualization of candidate proteins interacting with Zdhhc14 identified by IP-MS

Figure S3 Ubiquitination level of GBP5 with K48R and K63R mutants in mouse DRG tissues

Figure S4 Molecular dynamics simulations validate the binding interfaces between Zdhhc14 and GBP5.

(A) Root mean square deviation (RMSD) trajectories of Zdhhc14 (black), GBP5 (red), and the Zdhhc14–GBP5 complex (blue) over a 100 ns simulation. (B) Root mean square fluctuation (RMSF) profiles of Zdhhc14 and GBP5 residues. (C) The number of intermolecular hydrogen bonds formed between Zdhhc14 and GBP5 during the simulation. (D) Per-residue energy decomposition analysis identifying key residues contributing to the binding affinity.

Figure S5 GBP5 knockdown reverses the promotion of Zdhhc14 overexpression on L-OHP-induced pyroptosis in primary DRG neurons

(A) The protein levels of GBP5 in primary DRG neurons after overexpression detected by western blot. (B) LDH release rate of primary DRG neurons. (C-D) IL-18 and IL-1β levels in primary DRG neurons detected by ELISA. (E) Representative flow cytometry plots and (F) quantitative analysis of pyroptosis rate. (G) Protein expression levels of NLRP3, GSDMD-N, and CL-CASP1 in primary DRG neurons detected by Western blot. ***P<0.001 and ****P<0.0001.
